# Arming oncolytic herpes simplex virus with CXCL-11, IL-12 and a single-chain antibody against PD-1 to enhance CAR-T cell therapy in pancreatic ductal adenocarcinoma

**DOI:** 10.1038/s41419-026-08695-0

**Published:** 2026-04-04

**Authors:** Xixi Chen, Wenbo Ma, Yude Guan, Xiaohan Jin, Jingxuan Yu, Shumin Zhang, Nianchao Zhang

**Affiliations:** 1 Institute of Basic Medicine, North Sichuan Medical College, Nanchong, China; 2 School of Psychiatry, North Sichuan Medical College, Nanchong, China; 3 Key Laboratory of Microbial Functional Genomics of the Ministry of Education, College of Life Sciences, Nankai University, Tianjin, China

**Keywords:** Cancer immunotherapy, Drug discovery

## Abstract

Pancreatic ductal adenocarcinoma (PDAC), the primary form of pancreatic cancer, has a very poor prognosis and urgently requires effective treatments. Chimeric antigen receptor T (CAR-T) cell therapy presents a potential treatment approach, yet it is often hindered by several factors, including an immunosuppressive microenvironment, limited tumour infiltration, modest anti-tumour activity and short-term T cell persistence. Here, we engineered an oncolytic herpes simplex virus expressing CXCL-11, IL-12 and a single-chain antibody against PD-1 (named oHSV30) to enhance CAR-T cell infiltration, cytotoxicity and persistence in the tumour microenvironment, thereby improving its therapeutic efficacy. In both immunocompetent and immunodeficient syngeneic PDAC models, we demonstrated that the combination of CAR-T cells with the intratumoural administration of oHSV30 significantly reduced tumour burden and prolonged the survival of tumour-bearing mice. Overall, our data suggest that oHSV30 can be a promising adjuvant for CAR-T therapy in PDAC.

## Introduction

Pancreatic ductal adenocarcinoma (PDAC) is one of the most malignant cancers worldwide; once diagnosed, it is usually at an advanced stage and the 5-year survival rate is about 11% [1]. Chimeric antigen receptor T (CAR-T) cells have demonstrated remarkable clinical anti-tumour effects in hematological malignancies but have largely failed to achieve robust and durable responses against solid tumours [2]. The main limitations of CAR-T cell therapy that need to be addressed include poor tumour infiltration, limited efficacy and short persistence. Several mechanisms hinder the effectiveness of CAR-T therapy in solid tumours [3].

Oncolytic viruses are currently being considered as a potential therapeutic approach for the treatment of solid tumours based on a dual mechanism in which oncolytic viruses selectively target cancer cells, resulting in lysis of infected cells and induction of a systemic anti-tumour immune response [4]. Concurrently, the development of engineered oncolytic viruses carrying immunomodulatory agents has shown promise in significantly enhancing anti-tumour immunity [5]. Specifically, the inadequate infiltration of tumour-specific T cells into solid tumours, potentially due to suboptimal chemokine gradients, may be mitigated by the overexpression of C-X-C motif chemokine ligand 11 (CXCL-11) by oncolytic viruses, which has been correlated with increased CD8^+^ T cell infiltration and contributes to anti-tumour activity [6]. Additionally, Interleukin-12 (IL-12) has been shown to promote T-cell cytolysis and to stimulate the production of interferon (IFN)-γ, both of which are critical for an effective immune response against cancer [7]. Oncolytic viruses armed with IL-12 have been demonstrated to elicit potent anti-tumour activity by inducing an immunomodulatory effect within the tumour microenvironment (TME) [8–10]. Furthermore, immune checkpoint pathways, such as PD-1 and CTLA-4, can inhibit T cell expansion and persistence [11, 12]. The interaction between PD-1 expressed on CAR-T cells and its ligand PD-L1 expressed on tumour cells is recognised as a primary cause of non-response or weak response to CAR-T cell therapy [13]. Several studies have demonstrated that PD-1 blockade achieves greater persistence of CAR-T cells in preclinical mouse models and patients in clinical studies [13, 14].

In this study, CXCL-11 demonstrated stronger chemotactic activity for CAR-T cells than other chemokines. To address the aforementioned challenges to CAR-T cell efficacy in solid tumours, we engineered an oncolytic herpes simplex virus that co-expresses the chemokine CXCL-11, the cytokine IL-12 and a single-chain antibody against PD-1 (α PD-1 scFv), designated oHSV30. Intratumoural injection of oHSV30 significantly enhanced CAR-T cell infiltration, cytolytic activity and persistence, leading to effective suppression of established syngeneic PDAC in both immunocompetent and immunodeficient tumour-bearing mice. Our results show that oHSV30 treatment promotes PDAC eradication by CAR-T cells, providing a theoretical foundation for the clinical application of an oncolytic virus (OV)-based bioenhancer to augment the efficacy of CAR-T cells in solid tumours.

## Method and materials

### Cell lines and viruses

HEK293T and Vero cells, originally procured from the American Type Culture Collection (ATCC), were cultured in Dulbecco’s Modified Eagle Medium (DMEM, Gibco, 12800-017) supplemented with 10% Fetal Bovine Serum (FBS) and 1% penicillin/streptomycin. The replication of HSV-1-based oncolytic viruses was restricted in the C57BL/6 mice-derived pancreatic ductal adenocarcinoma (PDAC) cell line, Panc2. The oncolytic virus-susceptible and CAR-T cell-targetable cell line, Panc2-mCD19-HVEM, was established through lentiviral transduction of Panc2 cells using the pCDH-CMV-MCS-EF1-Puro vector, which encodes murine CD19 and human Herpesvirus Entry Mediator (mCD19-P2A-HVEM). To generate human CD19-expressing Panc1 cell lines, Panc1 cells were transduced with lentivirus expressing CD19 (pCDH-CMV-CD19-EF1-Puro). A clonally derived line was then maintained in medium containing 1.25 μg/mL puromycin. Incubation of all cells was conducted at 37 °C in a humidified atmosphere with 5% CO_2_. All the cell lines were characterized by STR analysis and were free of mycoplasma contamination.

The oncolytic viruses oHSV1, oHSV11 and oHSV12 were generated in our previous work [6]. oHSV-α PD-1, oHSV-h11, oHSV-h12 and oHSV-α hPD-1 were obtained by replacing the GFP cassette with the α PD-1 scFv (clone 332.8H3), human CXCL-11, human IL-12 and α human PD-1 scFv (clone RMP1-14) cassette in oHSV1 [13, 15]. oHSV30 and oHSV-h30 were obtained by inserting the IL-12 and α PD-1 scFv or human IL-12 and α human PD-1 scFv cassettes into the intergenic regions of UL3/UL4 and UL26/UL27 of oHSV11 or oHSV-h11, with the assistance of the CRISPR/Cas9 system. Growth and titration of all constructed oncolytic viruses were performed on Vero cells. Prior to in vitro and in vivo studies, all HSV-1-based oncolytic viruses were characterized as previously described [6].

### CAR vector construction and transduction of murine T cells

The CD19-targeted CAR construction includes murine CD8α signal peptide, murine CD19-targeted scFv, hinge, murine CD8 transmembrane region, the costimulatory domain of murine CD28 and CD3ζ and murine Thy1.1 was co-expressed to detect the CAR-transduced T cells. For the anti-mCD19 CAR construct, the DNA sequence was obtained from a published report and synthesised by Tsingke Biotechnology (Chengdu). Retroviruses were packaged by cotransfecting PlatE cells with the indicated plasmid and the helper plasmid pCL-Eco using calcium phosphate precipitation-mediated transfection. The viral supernatant was collected at 48 and 96 h after transfection, filtered through 0.45 µm filters, aliquoted and frozen at –80 °C.

Mouse T cells were purified from peripheral lymph nodes and spleens and activated with 10 μg/ml anti-CD3 (BioXCell; 145-2C11) and 5 µg/ml anti-CD28 (BioXCell; 37.51) overnight. Twenty-four h after activation, viral transduction was performed by spin-infection at 1500 g for 1 h at 30 °C in the presence of 16 µg/ml polybrene. Subsequently, the cells were washed and cultured in fresh TCM with IL-2 (2 ng/ml). Twenty-four h after spin infection, the efficiency of transduction was determined by examining reporter-positive cells (Thy1.1) using flow cytometry.

### CAR vector construction and transduction of human T cells

The construction of the CD19-targeted chimeric antigen receptor (CAR) included the CD8α signal peptide, a CD19-targeted scFv, a hinge region, the CD8 transmembrane domain, the costimulatory domain of human CD28 and the CD3ζ signalling domain. The DNA sequence of the anti-human CD19 CAR construct was derived from a published report and synthesised by Tsingke Biotechnology [16]. Lentiviruses were packaged by co-transfecting HEK293T cells with the plasmid encoding the CD19-targeted CAR, along with pMD2.G and psPAX2, using PEI transfection. Viral supernatant was collected 48 h post-transfection. The supernatants were filtered through 0.45 µm filters and the virus present in the supernatant was concentrated by ultracentrifugation at 25,000 rpm for 2 h. The concentrated virus was aliquoted and stored at –80 °C.

Peripheral blood mononuclear cells (PBMCs) were isolated from fresh whole blood obtained from healthy donors through gradient centrifugation using Ficoll-Paque PLUS (GE Healthcare; 17-1440-02). For T cell activation, 24-well plates were precoated with 10 μg/ml anti-hCD3 (BioXCell; UCHT1), 5 μg/ml anti-hCD28 (BioXCell; CD28.2). PBMCs were then seeded in these wells containing human T cell media, which was RPMI1640 medium supplemented with 10% FBS, 55 μM β-mercaptoethanol, 100 U/ml penicillin, 100 μg/ml streptomycin, 10 ng/ml human interleukin-2 (hIL-2). The culture was maintained in a humidified incubator at 37 °C with 5% CO_2_. After 24 h, activated T cells were infected with lentivirus expressing the CAR. The cells were expanded in human T cell media for an additional 3 days before proceeding to analysis or transfer into mice. The collection of peripheral blood samples from healthy donors was approved by the ethics committee for biomedical studies at North Sichuan Medical College and all volunteers involved in this study provided written consent.

### ELISA assay

For the ELISA assay, 1 × 10^5^ 293T cells or tumour cells were seeded in respective wells and infected with oHSV1, oHSV11, oHSV12, oHSV-α PD-1 scFv, or oHSV30 at a multiplicity of infection (MOI) of 1. After a 2-h incubation period, the infection medium was aspirated and fresh medium was added. Supernatants were collected from each group at 48 h post-infection. The concentrations of CXCL-11, IL-12 and α PD-1 scFv (Myc-Tag) protein in the supernatants were determined using specific ELISA kits. A similar approach was employed to assess the production of IFN-γ in CAR-T cells in coculture experiments. For the detection of in vivo chemokine (CXCL-11), cytokine (IL-12), or α PD-1 scFv expression, tumour homogenates were harvested from tumour-bearing mice 48 h following the administration of oncolytic virus for ELISA analysis.

### Chemotaxis assay

Migration assays were conducted in 24-well plates equipped with 5 μm pore size filters (Corning). Tumour cells (5 × 10^4^) infected with oncolytic viruses (oHSV1, oHSV11, or oHSV30) at a multiplicity of infection (MOI) of 1 for 48 h or media containing various recombinant chemokines at graded concentrations (0, 5, 10, 20 ng/ml) were placed in the bottom wells. Subsequently, CAR-T cells (1 × 10^6^) were added to the upper chambers. After 5 h of incubation, the cells that had migrated to the lower chambers were counted to assess and compare the differences in CAR-T cell migration.

### Migration cytotoxicity assay

To assess the cytotoxicity in a migration assay combining oncolytic virus and CAR-T cells in vitro, 5 × 10^4^ Panc2-mCD19-HVEM tumor cells were seeded in the lower chamber of a Transwell plate and incubated at 37 °C. After 24 h, the cells were treated with either PBS or the indicated oncolytic virus at an MOI of 1. Following 24 h of viral infection, 1 × 10^6 ^T cells were added to the upper chamber and incubated for an additional 4 h. Afterwards, the upper chamber containing the non-migrated T cells was removed and the migrated T cells in the lower chamber were further co-cultured with the tumor cells for 24 h. Tumor cell killing was assessed by measuring cell numbers using flow cytometry.

### Cytotoxicity assay

To determine the cell killing capacity of the combination of oncolytic virus and CAR-T cells in vitro, 5 × 10^4^ Panc2-mCD19-HVEM tumour cells were seeded in 24-well plates and incubated at 37 °C. After 24 h, the cells were treated with either PBS or the indicated oncolytic virus at an MOI of 1, followed by co-culturing with 1 × 10^5^ CAR-T cells for 48 h. To assess the cytotoxicity of tumour-infiltrated CAR-T cells, 2 × 10^4^ Panc2-mCD19-HVEM tumour cells were co-cultured with 1 × 10^5^ tumour-infiltrated CAR-T cells or CAR-T cells which were isolated from an equivalent tumour volume in a 96-well flat-bottom plate (Corning) for a duration of up to 36 h. The extent of target cell lysis was quantified using the CytoTox 96 Non-Radioactive Cytotoxicity Assay (Promega).

### Dissociation of single cells from tumour tissues

Tumour tissues were harvested from mice on the indicated days, minced and treated with dissociation solution (1 mg/mL collagenase I, 0.5 mg/mL DNase, dissolved in PBS) for 1 h at 37 °C with gentle shaking. After passage through 40 µm cell filters, the cells were washed with RPMI-1640/2% FBS.

### Preparation of tumour lymphocytes

Mice were inoculated with Panc2-mCD19-HVEM cells on day 0 and treated intratumourally with PBS or oncolytic viruses on days 6, 9 and 12 and received an intravenous injection of CAR-T cells (2 × 10^6^) on day 6. Tumour tissues were harvested from mice on day 21 after inoculation. Tumour-bearing mice were sacrificed on day 21 the indicated days and single cells of tumour tissue were resuspended in 40% Percoll gradient medium and overlaid with 70% Percoll. The suspension was centrifuged at 1260 × *g* for 20 min (25 °C) without interruption. Cells in the interface between the 40% and 70% Percoll were used as lymphocytes. The frequencies of CAR-T cells were determined with fluorescence-conjugated antibodies and analyzed by flow cytometry. The fluorescence-conjugated antibodies used in this study were listed as follows: Thy1.1-FITC (Biolegend; 202503), CD3-PE (Biolegend; 100205).

To measure cytokine production, isolated CAR-T cells were incubated at 37 °C for 3 h in 96-well flat-bottom plates after coculture with tumour cells, in the presence of 50 ng/ml phorbol 12-myristate-13-acetate (Sigma‒Aldrich; P8139), 500 ng/ml ionomycin (Sigma‒Aldrich; I0634) and 1 μg/ml brefeldin A (Sigma‒Aldrich; B6542). After surface staining, the Fixation/Permeabilization Solution Kit (BD; 554714) was used for cytokine detection. Fluorescently conjugated antibodies against cell-surface, intracellular and nuclear antigens were used as follows: Thy1.1-FITC (Biolegend; 202503), CD3-PE (Biolegend; 100205) and IFN-γ-FITC (Biolegend; 505806). Flow cytometric analysis was performed on a CytoFlex (Beckman Coulter) using FlowJo software.

### Cell proliferation assay

Panc2-mCD19-HVEM cells were cultured in 96-well plates at a density of 5 × 10^4^ cells per well. They were then infected with the indicated viruses at an MOI of 1, 24 h after cell inoculation. At the indicated time points, CCK-8 reagent (Cell Counting Kit-8, Solarbio, CA1210) was added to the cells and incubated for 1 h at 37 °C. Absorbance at 450 nm was measured using a microplate reader (Synergy 4; BioTek). The percentage of cell viability was calculated as follows: (OD value of infected cells–OD value of blank)/(OD value of uninfected cells–OD value of blank) × 100%.

### RT-qPCR analysis

Total RNA was extracted using Trizol reagent (Invitrogen, USA). cDNA was synthesised using the Reverse Transcription System kit (Vazyme; USA) and gene expression analysis was performed using the Biosin system. The relative mRNA amounts of genes of interest to endogenous control β-actin were presented using arbitrary units. Primer information is listed in the Supplementary Table S1.

### Animal experiments

Animal experiments were performed in accordance with the Institutional Animal Care and Use Committee of North Sichuan College. C57BL/6 mice were purchased from Beijing Vitonglihua Laboratory Animal Science and Technology Co., Ltd. Rag1^–/–^ mice (JAX: 034159), CD4^Cre^ mice (JAX: 022071), CXCR3^fl/fl^ mice (JAX:005796) and NSG mice (JAX: 005557) originally came from The Jackson Laboratory. These mice were housed in an SPF environment with a 12 h light/ dark cycle and provided with a standard laboratory rodent diet and water. For the treatment experiments, sex and age-matched C57BL/6 J mice or Rag1^–/–^ mice were implanted with Panc2-mCD19-HVEM cells (1 × 10^5^ or 5 × 10^4^ cells) in the right flank of each mouse. For the xenograft tumour model, 1 × 10^6^ Panc-1 tumour cells were injected subcutaneously into NSG mice. When the tumour was established (6 days after tumour injection), mice were randomly divided into different groups (six mice per group). Oncolytic viruses (1 × 10^7^ PFU per mouse) were diluted in PBS and viruses or PBS were administered intratumourally on days 6, 9 and 12. For combination therapy or CAR-T monotherapy, CAR-T cells (2 × 10^6^ cells per mouse) were injected via the tail vein on day 6. Tumour volume (mm^3^) was measured with a caliper for 3 days and calculated as (length × width^2^)/2. For survival analysis, mice with tumours larger than the volume limit of 1500 mm^3^ were sacrificed and counted as dead. Tumour-free survival of the treated mice was assessed during a post-treatment monitoring period of more than 60 days.

### Statistical analysis

All quantitative data were expressed as mean ± SD. Statistical analysis was calculated with GraphPad Prism 10 software. Student’s *t* test was utilised to compare two independent groups, and a one-way ANOVA model with the correction of Tukey was utilised to compare three or more groups. A two-way ANOVA followed by Tukey’s post hoc test was used when groups were considered as independent factors. Animal survival times were presented by the Kaplan–Meier method, and the *p-*values were calculated and compared with the log-rank test. The samples and cells were randomised into different groups prior to treatment. All samples and animals were included in the final analysis and all evaluation criteria were set in advance. Sample sizes for the experiments were not predetermined by any statistical method, nor were they selected based on statistical power considerations to detect a predefined effect size. P values of < 0.05 were statistically significant (*p < 0.05, **p < 0.01, ***p < 0.001 and ****p < 0.0001).

## Results

### CXCL-11 is a superior chemoattractant for CAR-T cells

**Fig. 1:**
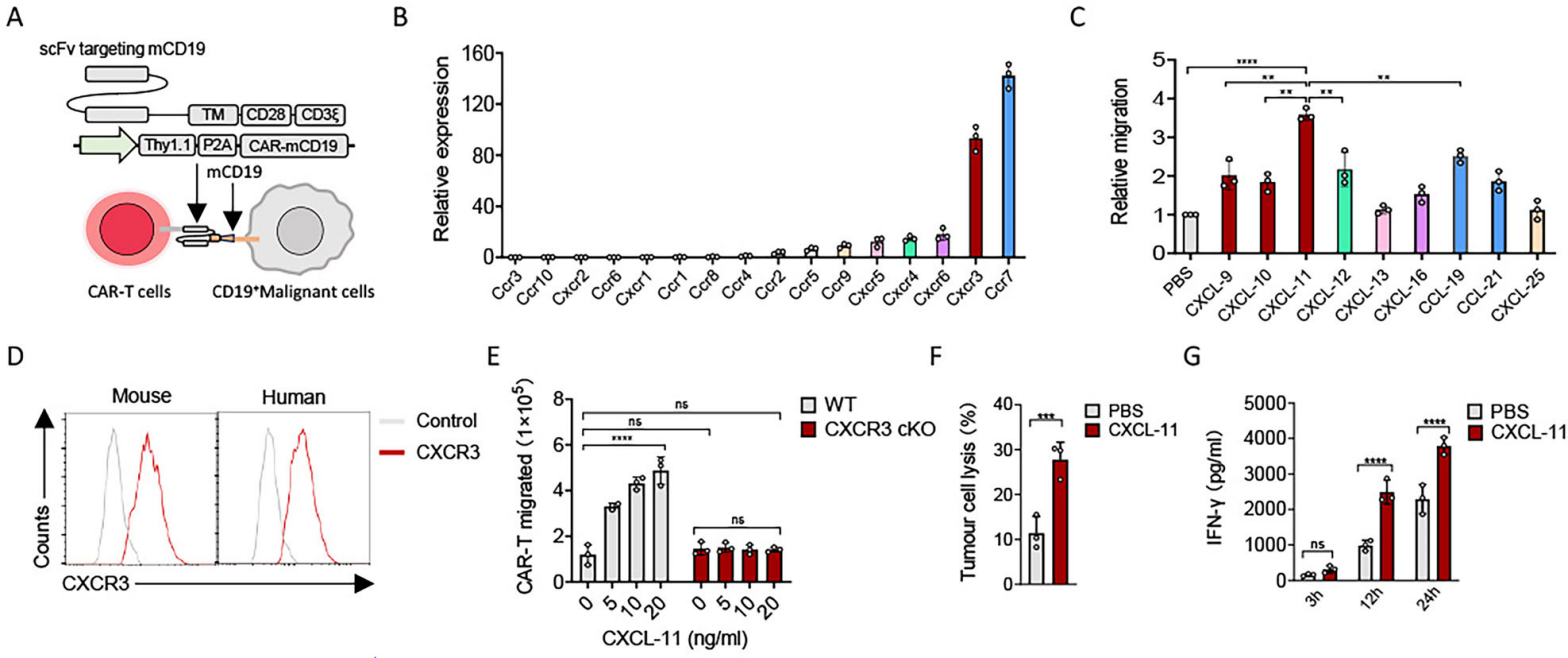
CXCL-11 are potent ligands to attract CAR-T cells. **A** The composition of the anti-mCD19 CAR. **B** The expression of chemokine receptors in murine CAR-T cells was quantified by quantitative PCR (*n* = 3). **C** The migration of CAR-T cells was evaluated by adding different kinds of T-cell chemotactic factor using a transwell migration assay (*n* = 3 experiments). **D** The expression of CXCR3 on murine and human CAR-T cells was measured by flow cytometry. **E** The transwell migration of CAR-T cells and CXCR3-deficient CAR-T cells towards increasing concentrations of recombinant murine CXCL-11 was assessed. After 3 h, the number of migrated T cells was quantified by flow cytometry (*n* = 3 experiments). CAR-T cells (2 × 10^5^) were plated to the upper chambers and the lower chambers contained Panc2-mCD19-HVEM (5 × 10^4^) supplemented with CXCL-11 or saline control. **F** Lysis of Panc2-mCD19-HVEM by CAR-T cells following migration through a permeable membrane. After a migration period of 3 h, migrated T cells and tumour cells were co-cultured for a further 48 h. **G** ELISA revealing time-dependent activation levels of migrated CAR-T cells upon co-culture with Panc2-mCD19-HVEM tumour cells (*n* = 3 experiments). Data represent the mean ± SD. Statistical significance was determined by two-sided unpaired t-test (**F**), one-way ANOVA with Tukey’s significant difference multiple comparisons (**C**) and two-way ANOVA with Sidak’s significant difference multiple comparisons (**E**, **G**). ns not significant, *p < 0.05, **p < 0.01, ***p < 0.001, ****p < 0.0001.

To investigate the anti-tumour efficacy of CAR-T cells assisted by an oncolytic virus on PDAC, we employed a previously published CD19 CAR targeting mouse CD19 (mCD19) and an HSV-based oncolytic virus [6, 17]. While human PDAC cell lines were susceptible to HSV-based oncolytic viruses, viral replication was notably restricted in the C57BL/6 mouse-derived PDAC cell line Panc02. Thus, we generated an HSV-1-based oncolytic virus-infectible and CAR-T cell-targetable cell line stably expressing murine CD19 and human herpes virus entry mediator (HVEM), named Panc2-mCD19-HVEM (Fig. 1A).

The infiltration of CAR-T cells into the tumour microenvironment (TME) is crucial for the anti-tumour immune response and this process largely depends on the interaction between their chemokine receptors and chemokines. To identify suitable targets for recruiting CAR-T cells to tumours, we analyzed the RNA expression levels of chemokine receptors in both murine and human CAR-T cells and found that CCR7, CXCR3, CXCR6 and CXCR4 was higher in both models (Figs. 1B and S1A). Given the key role of chemokine receptors in facilitating T cell infiltration, we then attempted to find an ideal chemokine to attract them. A Transwell migration assay was used to evaluate the chemotactic activity of several chemokines. The data showed that chemokine CXCL-11 promoted more CAR-T cells to migrate into the lower chamber (Fig. 1C). CXCL-11, a ligand for CXCR3, exhibits potent anti-tumour activity by recruiting cytotoxic T lymphocytes (CTLs) and T helper 1 (Th1) cells into the TME; these cells predominantly exhibit cytotoxicity against tumour cells [18]. In line with this, flow cytometry analysis confirmed that CAR-T cells expressed high levels of CXCR3 (Fig. 1D). Moreover, we observed that CAR-T cells, but not CXCR3-deficient CAR-T cells, migrated towards CXCL-11 in a dose-dependent manner, suggesting that CXCR3 expression is crucial for this chemotactic response (Figs. 1E and S1B–D). By combining migration and cytotoxicity assays, we found that CXCL-11 promoted the migration of CAR-T cells towards Panc2-mCD19-HVEM cells, which resulted in enhanced target cell lysis compared with the PBS-treated group (Fig. 1F) and accelerated CAR-T cell activation (Fig. 1G). These results suggest that CXCL-11 acts as a potent chemoattractant for CAR-T cells, implying that increasing its expression could enhance the anti-tumour effect at the tumour site.

### oHSV11 enhances CAR-T cell anti-tumour efficacy in PDAC by promoting migration and tumour infiltration

**Fig. 2:**
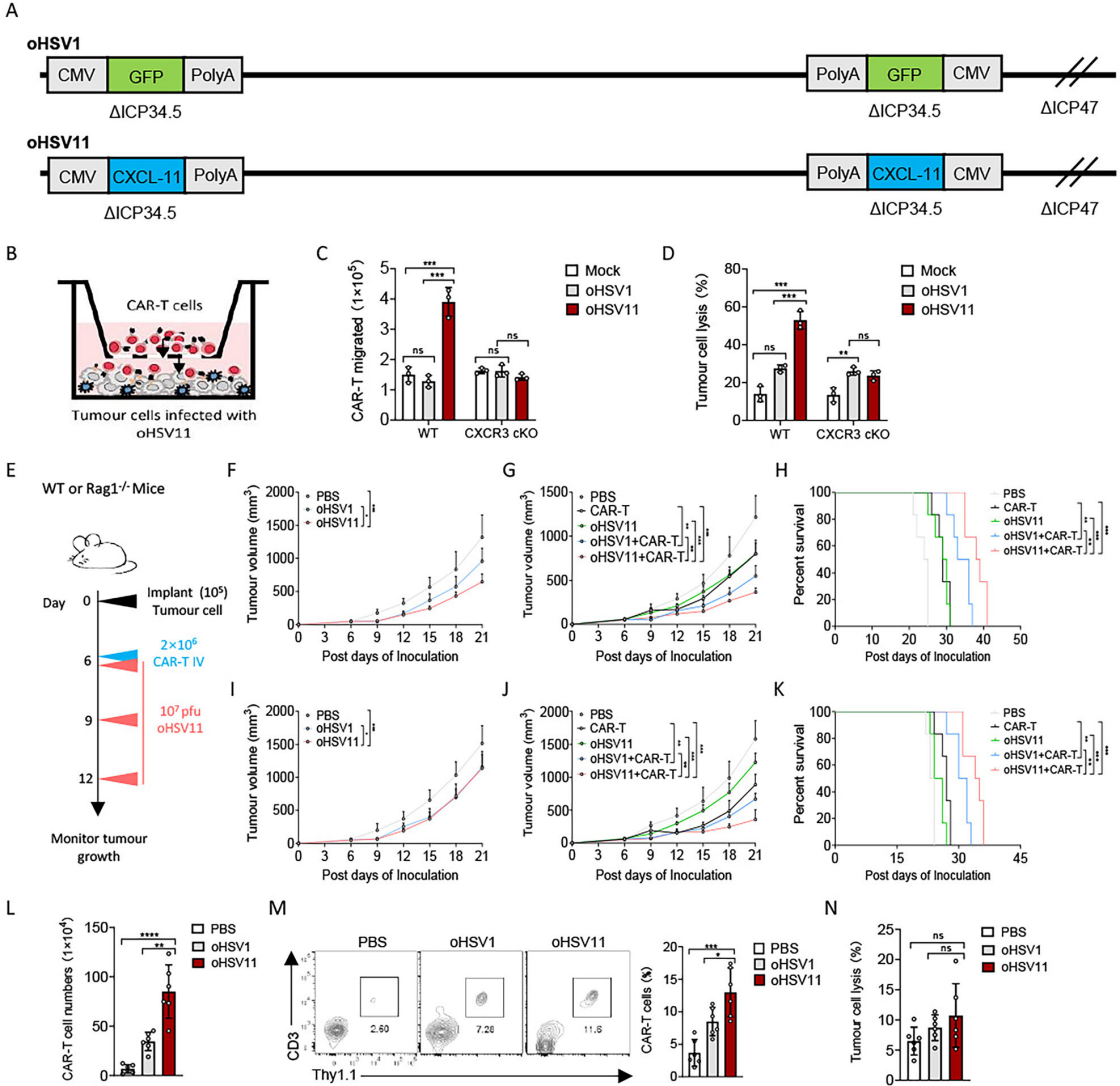
oHSV11 improves the PDAC tumour control ability of CAR-T therapy. **A** Scheme showing the construction of oncolytic herpes simplex viruses expressing CXCL-11 (oHSV11). **B** Schema of the migration cytotoxicity assays based on the transwell assay. CAR-T cells (1 × 10^6^) were plated in the upper chambers and the lower chambers contained Panc2-mCD19-HVEM (5 × 10^4^) infected with oHSV11, oHSV1, or saline control. **C**, **D** The migration of CAR-T cells or CXCR3-deficient CAR-T cells and the number of tumour cells were analyzed by flow cytometry (*n* = 3 experiments). **E**–**K** Experimental scheme. **F** Mice on a C57BL/6J background were implanted with Panc2-mCD19-HVEM (1 × 10^5^) cells on day 0 and treated intratumourally with oncolytic viruses or PBS on days 6, 9 and 12. Tumour progression was measured every 3 days after the first treatment (*n* = 6 per group). **G**, **H** Panc2-mCD19-HVEM tumour-bearing mice were treated intratumourally with oncolytic viruses or PBS on days 6, 9 and 12 and received an intravenous injection of PBS or CAR-T cells (2 × 10^6^) on day 6 (*n* = 6 per group). Tumour progression was measured every 3 days after the first treatment. A Kaplan-Meier survival curve was used to analyze the survival of tumour-bearing mice treated with oncolytic viruses. **I** Rag1^–/–^ mice were implanted with Panc2-mCD19-HVEM (5 × 10^4^) cells on day 0 and treated intratumourally with oncolytic viruses or PBS on days 6, 9 and 12. Tumour volume was measured every 3 days after the first treatment (*n* = 6 per group). Panc2-mCD19-HVEM tumour-bearing Rag1^–/–^ mice were treated intratumourally with oncolytic viruses or PBS on days 6, 9 and 12 and received an intravenous injection of PBS or CAR-T cells (2 × 10^6^) on day 6 (n = 6 per group). Tumour progression (**J**) and survival curve (**K**) were measured on the indicated days. Panc2-mCD19-HVEM tumour-bearing Rag1^–/–^ mice were treated intratumourally with indicated oncolytic viruses or PBS on days 6, 9 and 12 and received an intravenous injection of CAR-T cells (2 × 10^6^) on day 6. **L**–**N** Tumour tissues were harvested from mice on day 21 after inoculation (*n* = 6 per group). **L** Tumour-infiltrating T cells were analyzed by flow cytometry. **N** Panc2-mCD19-HVEM tumour cells were co-cultured with tumour-infiltrating CAR-T cells (1 × 10^5^) in a 96-well flat-bottom plate. The lysis of tumour cells was measured after 36 h. Data represent the mean ± SD. Statistical significance is calculated by one-way ANOVA with Tukey’s significant difference multiple comparisons (**F**, **G**, **I**, **J**, **L**–**N**) or two-way ANOVA with Tukey’s significant difference multiple comparisons (**C**, **D**). ns not significant, *p < 0.05, **p < 0.01, ***p < 0.001, ****p < 0.0001.

CXCL-11 promotes the migration of CAR-T cells to tumour sites, which has been shown to enhance their cancer cell-killing ability. To evaluate the combined therapeutic effect of CXCL-11-expressing oncolytic virus and CAR-T cells, we constructed an oncolytic herpes simplex virus (oHSV11) that expresses CXCL-11 and assessed its efficacy against PDAC in combination with CAR-T cells (Fig. 2A). As shown in Fig. 2B, we assessed the combined therapeutic effect of oHSV11 and CAR-T cells on PDAC using a migration cytotoxicity assay. Panc2-mCD19-HVEM cells were first infected for 24 h with oHSV1, oHSV11, or a saline control. CAR-T cells or CXCR3-deficient CAR-T cells were then added to the upper chambers of a transwell system. After a further 24 h, flow cytometry analysis revealed that infection with oHSV11, but not with the parental oHSV1, enhanced both the migration of CAR-T cells and the cytolysis of tumour cells. In contrast, oHSV11 failed to promote the migration of CXCR3-deficient CAR-T cells. Consequently, the loss of this migratory response abolished the enhanced anti-tumour efficacy of the oHSV11 and CAR-T cell combination (Fig. 2C, D).

To evaluate the anti-tumour efficacy of the oHSV11 and CAR-T cell combination against PDAC in vivo, we established subcutaneous Panc2-mCD19-HVEM tumours in immunocompetent and immunodeficient mice (Fig. 2E). First, the oncolytic potency of the oHSV11 was assessed in immunocompetent mice. Administration of oHSV11 on days 6, 9 and 12 suppressed tumour growth compared with the parental oHSV1 (Fig. 2F). In combination studies, co-administration of oHSV11 and CAR-T cells resulted in a significantly reduced tumour growth rate and prolonged survival relative to all other treatment groups (oHSV11 alone, CAR-T cells alone or oHSV1 plus CAR-T cells) in immunocompetent hosts (Fig. 2G, H). We next examined the combination in Rag1^–/–^ mice to exclude adaptive immune interference. While oHSV11 alone did not show superior tumour control versus oHSV1 in this setting, the combination of oHSV11 with CAR-T cells significantly suppressed tumour progression and extended survival compared with monotherapies or the oHSV1-based combination (Fig. 2I–K). Flow cytometric analysis of tumour infiltrates after three viral doses revealed that oHSV11 treatment markedly enhanced CAR-T cell accumulation within tumours compared with CAR-T cell monotherapy or oHSV1 plus CAR-T cells (Fig. 2L, M). However, in vitro cytotoxicity assays of tumour-infiltrating CAR-T cells showed no significant difference in cytolytic capacity between the combination groups receiving oHSV1 or oHSV11 (Fig. 2N). In summary, CAR-T cell monotherapy was ineffective against PDAC tumours. The enhanced anti-tumour efficacy of the combination therapy was driven by oHSV11, which facilitated tumour infiltration of CAR-T cells via the CXCL-11–CXCR3 axis rather than by boosting their cytotoxic activity.

### oHSV12 enhances CAR-T cell anti-tumour efficacy in PDAC by potentiating cytotoxicity

**Fig. 3:**
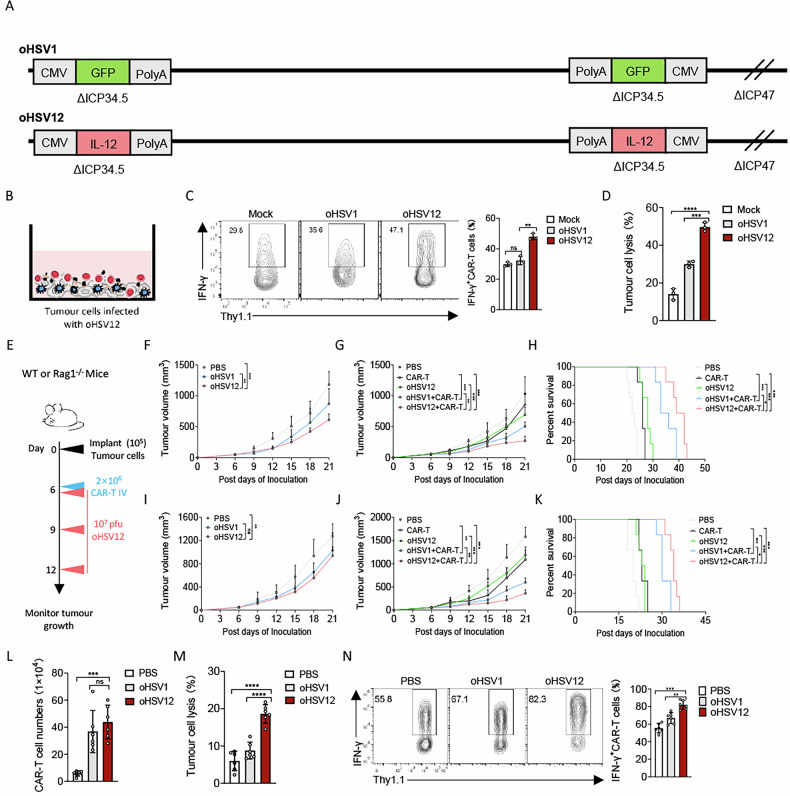
Combined oHSV12 and CAR-T cells have a superior cytotoxic effect against PDAC tumours in vivo. **A** Scheme showing the construction of oncolytic herpes simplex viruses expressing IL-12. **B** Schema of cytotoxicity assay. Tumour cells were cultured in plates for 48 h and infected with oHSV1 or oHSV12 at an MOI of 1. CAR-T cells were then co-cultured with the infected Panc2-mCD19-HVEM cells (*n* = 3 experiments). The IFN-γ production in CAR-T cells (**C**) and tumor cell lysis (**D**) were analyzed by flow cytometry. **E**–**K** Experimental scheme. Panc2-mCD19-HVEM tumour-bearing mice WT mice or Rag1^–/–^ mice received a single administration of oncolytic viruses or PBS on days 6, 9 and 12, or both three doses of oncolytic viruses (1 × 10^7^ pfu) and CAR-T cells (2 × 10^6^) on the indicated days (*n* = 6 per group). **F**, **G** Tumour progression was measured every 3 days after the first treatment with oncolytic viruses. **H** Survival times of tumour-bearing mice were analyzed using the Kaplan-Meier method with the log-rank test. **I** Rag1^–/–^ mice were implanted with Panc2-mCD19-HVEM cells (5 × 10^4^) on day 0 and treated intratumourally with oncolytic viruses or PBS on days 6, 9 and 12. Tumour volume was measured every 3 days after the first treatment (*n* = 6 per group). Panc2-mCD19-HVEM tumour-bearing Rag1^–/–^ mice were treated intratumourally with oncolytic viruses or PBS on days 6, 9 and 12 and received an intravenous injection of PBS or CAR-T cells (2 × 10^6^) on day 6 (*n* = 6 per group). Tumour progression (**J**) and survival curve (**K**) were measured on the indicated days. Panc2-mCD19-HVEM tumour-bearing Rag1^–/–^ mice were treated intratumourally with the indicated oncolytic viruses or PBS on days 6, 9 and 12 and received an intravenous injection of CAR-T cells (2 × 10^6^) on day 6. Tumour tissues were harvested from mice on day 21 after inoculation (*n* = 6 per group). **L** Tumour-infiltrating T cells were analyzed by flow cytometry. Panc2-mCD19-HVEM tumour cells were co-cultured with tumour-infiltrating CAR-T cells (1 × 10^5^) in a 96-well flat-bottom plate. The lysis of tumour cells (**M**) and the production of IFN-γ in CAR-T cells (**N**) were measured after 36 h. Data represent the mean ± SD. Statistical significance is calculated by one-way ANOVA with Tukey’s significant difference multiple comparisons (**C**, **D**, **F**, **G**, **I**, **J**, **L**–**N**). ns not significant, *p < 0.05, **p < 0.01, ***p < 0.001, ****p < 0.0001.

IL-12 is a pro-inflammatory cytokine known to augment T cell cytotoxicity and exert potent anti-tumour effects. To investigate whether localized IL-12 delivery via an oncolytic virus could enhance the efficacy of CAR-T cells against PDAC, we engineered an oncolytic herpes simplex virus expressing IL-12, named oHSV12 (Fig. 3A). In vitro cytotoxicity assays revealed that infection of PDAC cells with oHSV12 significantly enhanced the tumour-killing activity of co-cultured CAR-T cells (Fig. 3B, C). This potentiation correlated with increased IFN-γ production by CAR-T cells (Fig. 3D). These findings demonstrate that oHSV12-mediated local delivery of IL-12 robustly augments both cytotoxic function and cytokine production in CAR-T cells, highlighting a promising strategy to potentiate adoptive T-cell therapies against solid tumours.

We next evaluated the efficacy of this combination therapy in vivo using immunocompetent mice bearing Panc2-mCD19-HVEM tumours (Fig. 3E). In this model, monotherapy with oHSV12 moderately suppressed tumour growth relative to oHSV1 (Fig. 3F). The combination of oHSV12 with CAR-T cells, however, resulted in significantly greater tumour inhibition compared to either treatment alone or to oHSV1 plus CAR-T cells (Fig. 3G) and extended the survival of tumour-bearing mice (Fig. 3H). To dissect the contribution of the adaptive immune system, we turned to Rag1^–/–^ mice bearing PDAC tumours. In this immunodeficient setting, oHSV12 alone did not enhance tumour control beyond that of oHSV1 (Fig. 3I). By contrast, the combination of oHSV12 with CAR-T cells markedly slowed tumour progression and improved survival compared to oHSV1 plus CAR-T cells (Fig. 3J, K). Although tumour-infiltrating CAR-T cell numbers were comparable between the two combination groups, CAR-T cells in the oHSV12 cohort exhibited significantly higher cytotoxic activity (Fig. 3L, M), consistent with the known role of IL-12 in potentiating T-cell function. This enhanced cytotoxicity was further supported by elevated IFN-γ production in CAR-T cells from the oHSV12 combination group (Fig. 3N). Collectively, these results demonstrate that localized IL-12 delivery via oHSV12 potently enhances the in vivo cytolytic capacity of CAR-T cells, leading to superior tumour control and prolonged survival.

### oHSV-α PD-1 augments CAR-T cell therapy in PDAC through enhancement of persistence

**Fig. 4:**
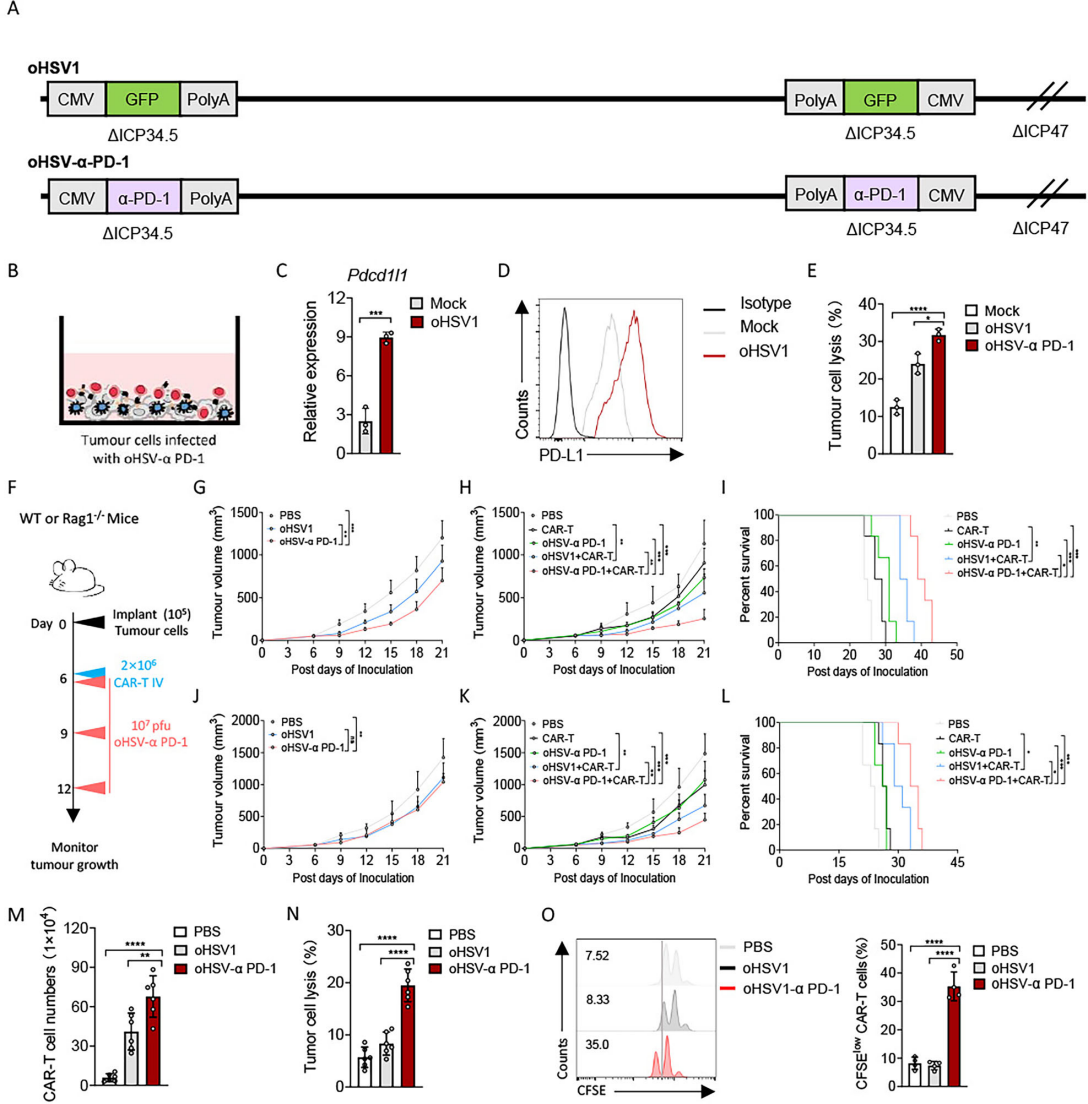
oHSV-α PD-1 enhanced the persistence of CAR-T therapy in vivo. **A** Scheme showing the construction of oncolytic herpes simplex viruses expressing α PD-1 scFv (oHSV-α PD-1). **B** Schema of cytotoxicity assay. Tumour cells were cultured in plates for 48 h and infected with oHSV1 or oHSV-α PD-1 at an MOI of 1. CAR-T cells were then co-cultured with the infected Panc2-mCD19-HVEM cells. The expression of Pdcd1l1 mRNA and PD-L1 protein was analyzed by quantitative RT-PCR (**C**) and flow cytometry (**D**) in Panc2-mCD19-HVEM cells infected with oHSV1 at an MOI of 1 for 48 h (*n* = 3 experiments). **F**–**L** Experimental scheme. Panc2-mCD19-HVEM tumour-bearing mice, WT mice or Rag1^–/–^ mice received a single administration of oncolytic viruses or PBS on days 6, 9 and 12, or both three doses of oncolytic viruses (1 × 10^7^ pfu) and CAR-T cells (2 × 10^6^) on the indicated days (*n* = 6 per group). **G**, **H** Tumour progression was measured every 3 days after the first treatment. **I** A Kaplan–Meier survival curve was used to analyze the survival of tumour-bearing mice treated with oncolytic viruses. **J** Rag1^–/–^ mice were implanted with Panc2-mCD19-HVEM (5 × 10^4^) on day 0 and treated intratumourally with oncolytic viruses or PBS on days 6, 9 and 12. Tumour volume was measured every 3 days after the first treatment (*n* = 6 per group). **K**, **L** Panc2-mCD19-HVEM tumour-bearing Rag1^–/–^ mice were treated intratumourally with oncolytic viruses or PBS on days 6, 9 and 12 and received an intravenous injection of PBS or CAR-T cells (2 × 10^6^) on day 6 (*n* = 6 per group). Tumour progression (**K**) and survival curve (**L**) were measured on the indicated days (*n* = 6 per group). Panc2-mCD19-HVEM tumour-bearing Rag1^–/–^ mice were treated intratumourally with the indicated oncolytic viruses or PBS on days 6, 9 and 12 and received an intravenous injection of CAR-T cells (2 × 10^6^) on day 6. Tumour tissues were harvested from mice on day 21 after inoculation (*n* = 6 per group). **M** Tumour-infiltrated T cells were analyzed by flow cytometry. **N**, **O** Panc2-mCD19-HVEM tumour cells were co-cultured with 1 × 10^5^ tumour-infiltrating CAR-T cells with CFSE staining in a 96-well flat-bottom plate. The lysis of tumour cells (**N**) and proliferation of CAR-T cells (**O**) were measured after 36 h. Data represent the mean ± SD. Statistical significance was determined by two-sided unpaired t-test (**C**) and one-way ANOVA with Tukey’s significant difference multiple comparisons (**E**, **G**, **H**, **I**–**K**, **M**–**O**). ns not significant, *p < 0.05, **p < 0.01, ***p < 0.001, ****p < 0.0001.

Immunotherapies such as immune checkpoint blockade can restore antitumour T cell effector functions, offering improved clinical outcomes in cancer patients [19]. In particular, immune checkpoint inhibitors, including α PD-1 mAbs, reinvigorate antitumour T cell activity and promote their persistence [20]. Building on evidence that oncolytic viruses engineered to secrete PD-1/PD-L1 inhibitors can elicit systemic T cell-mediated antitumour immunity [21], we constructed an oncolytic herpes simplex virus expressing an α PD-1 scFv (oHSV-α PD-1) and validated the combinational therapy with CAR-T cells (Fig. 4A, B). Initial analysis revealed that oncolytic virus infection upregulates PD-L1 expression on tumour cells (Fig. 4C, D). Compared with control oHSV1 treatment, oHSV-α PD-1 significantly enhanced the tumour-killing capacity of CAR-T cells (Fig. 4E).

To determine whether an oncolytic virus expressing an anti-PD-1 antibody could potentiate the antitumour efficacy of CAR-T cells in vivo, we tested the combination of oHSV-α PD-1 with CAR-T cells in a syngeneic PDAC model using both immunocompetent and immunodeficient mice (Fig. 4F). In immunocompetent mice, oHSV-α PD-1 monotherapy led to superior tumour control relative to oHSV1 (Fig. 4G). Moreover, the combination of oHSV-α PD-1 with CAR-T cells enhanced antitumour activity compared with either agent alone or with oHSV1 plus CAR-T cells (Fig. 4H, I). In T-cell-deficient Rag^–/–^ tumour-bearing mice, oHSV-α PD-1 alone did not suppress tumour growth beyond the effect of oHSV1; however, combining oHSV-α PD-1 with CAR-T cells resulted in stronger tumour inhibition and prolonged survival compared with all other groups (Fig. 4J–L). Flow cytometry revealed increased tumour infiltration of CAR-T cells in mice treated with oHSV-α PD-1 plus CAR-T cells relative to those receiving oHSV1 plus CAR-T cells (Fig. 4M), suggesting that the PD-1-blocking oncolytic virus promotes CAR-T cell persistence in tumours. Consistent with this, in vitro cytotoxicity assays and CFSE-based proliferation analysis showed that CAR-T cells isolated from the combination-therapy group exhibited enhanced cytolytic function and expanded proliferative capacity (Fig. 4N, O). Together, these data indicate that oHSV-α PD-1 confers a proliferative advantage to CAR-T cells within the tumour microenvironment, thereby amplifying their tumour-killing efficacy.

### Triple-armed oHSV30 (CXCL-11/IL-12/α PD-1 scFv) synergises with CAR-T cells to enhance in vitro cytotoxicity against PDAC

**Fig. 5:**
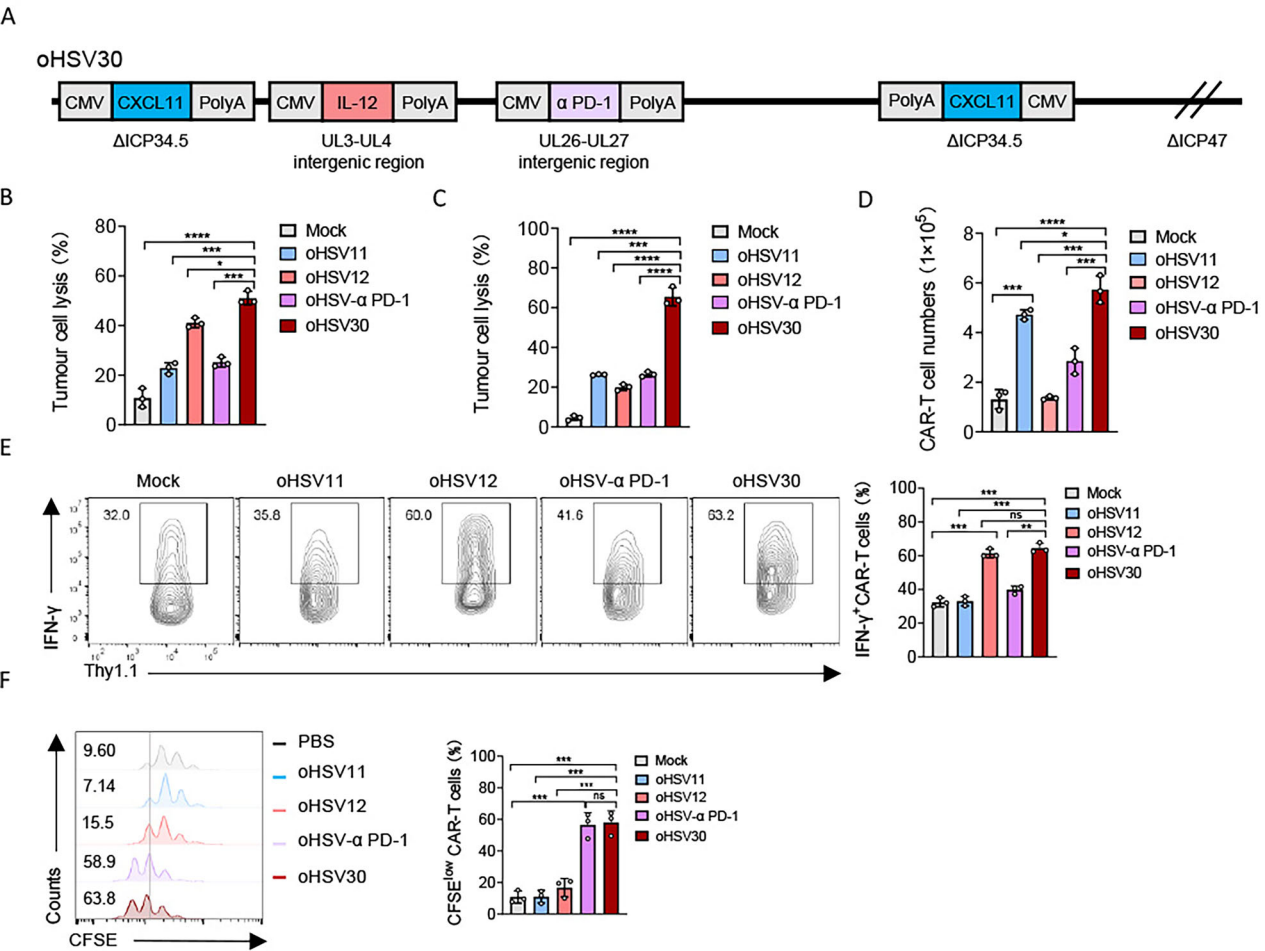
oHSV30 enhanced migration, lytic activity and persistence of CAR-T cells. **A** Scheme showing the construction of oncolytic herpes simplex viruses oHSV30. **B** Panc2-mCD19-HVEM cells were cultured in plates and infected with oHSV11, oHSV12, oHSV-α PD-1, or oHSV30 at an MOI of 1. CAR-T cells were co-cultured with the infected Panc2-mCD19-HVEM cells. The number of tumour cells was analyzed by flow cytometry (*n* = 3 experiments). **C**–**F** CAR-T cells (2 × 10^5^) were plated in the upper chambers and the lower chambers contained Panc2-mCD19-HVEM (5 × 10^4^) infected with oHSV11, oHSV12, oHSV-PD-1, oHSV30, or saline control. The lysis of tumour cells, the migration of CAR-T cells towards the lower chambers, IFN-γ production in CAR-T cells and the proliferation of CAR-T cells were analyzed by flow cytometry (*n* = 3 experiments). Data represent the mean ± SD. Statistical significance is calculated by one-way ANOVA with Tukey’s significant difference multiple comparisons (**B**–**F**). ns not significant, *p < 0.05, **p < 0.01, ***p < 0.001, ****p < 0.0001.

Since tumours have a complex immunosuppressive TME, a single therapeutic gene armed oncolytic virus may be insufficient to overcome the limitations to anti-tumour activity for CAR-T cells. On this basis, the use of oncolytic viruses delivering multiple immunomodulators will be a more potent approach to enhance immune cell-mediated anti-tumour immune responses. We identified two intergenic sites (between UL3-UL4 and UL26-UL27) suitable for exogenous gene insertion and confirmed through viral titration and plaque assays that integration at these loci did not compromise viral virulence (Fig. S2A–G). To simultaneously overcome these limitations of CAR-T cells described above, an oncolytic herpes simplex virus expressing CXCL-11, IL-12 and α PD-1 scFv (oHSV30) was constructed based on the backbone of oHSV11, aiming to improve the therapeutic efficacy of CAR-T cells in solid tumours (Fig. 5A). Infection of 293 T cells with oHSV30 confirmed that the expression of CXCL-11, IL-12 and α PD-1 scFv did not impair viral replication relative to the parental virus (Fig. S3A), while also yielding high levels of each immunomodulator (Fig. S3B–E). The oncolytic potency of oHSV30 remained intact, as evidenced by comparable losses in viability (CCK-8 assay) and similar induction of apoptosis in Panc1 cells infected with oHSV30 versus oHSV1 (Fig. S3F, G). Together, these data demonstrate that oHSV30 efficiently expresses and secretes multiple effector molecules without compromising its replicative or lytic capacity.

Previous studies have demonstrated that oncolytic viruses engineered to express CXCL-11, IL-12, or α PD-1 scFv can enhance CAR-T cell infiltration, cytotoxicity and persistence against solid tumours. We therefore sought to investigate whether the triple-armed oHSV30 (encoding CXCL-11, IL-12 and α PD-1 scFv) could further augment the anti-tumour efficacy of CAR-T cells in vitro. In a cytotoxicity assay, Panc2-mCD19-HVEM cells were infected with various oncolytic viruses and then co-cultured with CAR-T cells. Tumour cell lysis was most significantly enhanced in the oHSV30-infected group compared to groups infected with oHSV1, oHSV11, or oHSV-α PD-1 and showed a modest improvement over the oHSV12-infected group (Fig. 5B). A Transwell-based migration-cytotoxicity assay further confirmed that the combination of oHSV30 and CAR-T cells induced target tumour cell death more effectively than combinations using oHSV1, oHSV11, oHSV12, or oHSV-α PD-1 (Fig. 5C). Within this assay, we also assessed the effects of oHSV30 on CAR-T cell migration, polarization and proliferation. The number of CAR-T cells migrating to the lower chamber was significantly higher in the oHSV11 and oHSV30 groups compared to other viruses, with oHSV30 showing a minimal increase over oHSV11 (Fig. 5D). Furthermore, IFN-γ production in CAR-T cells was markedly increased in both the oHSV12 and oHSV30 groups, with comparable frequencies of IFN-γ^+^ CAR-T cells observed between these two conditions (Fig. 5E). These data support the role of virus-delivered IL-12 as a positive regulator of CAR-T cell cytotoxicity. Analysis of CFSE-pre-stained CAR-T cells revealed superior proliferative ability in the oHSV-α PD-1 and oHSV30 groups (Fig. 5G). In summary, these results indicate that oHSV30, armed with CXCL-11, IL-12 and α PD-1 scFv, confers comprehensive anti-tumour activity on CAR-T cells through multiple synergistic mechanisms, leading to enhanced tumour cell death in vitro.

### oHSV30 synergises with CAR-T cells to enhance anti-tumour response in PDAC

**Fig. 6:**
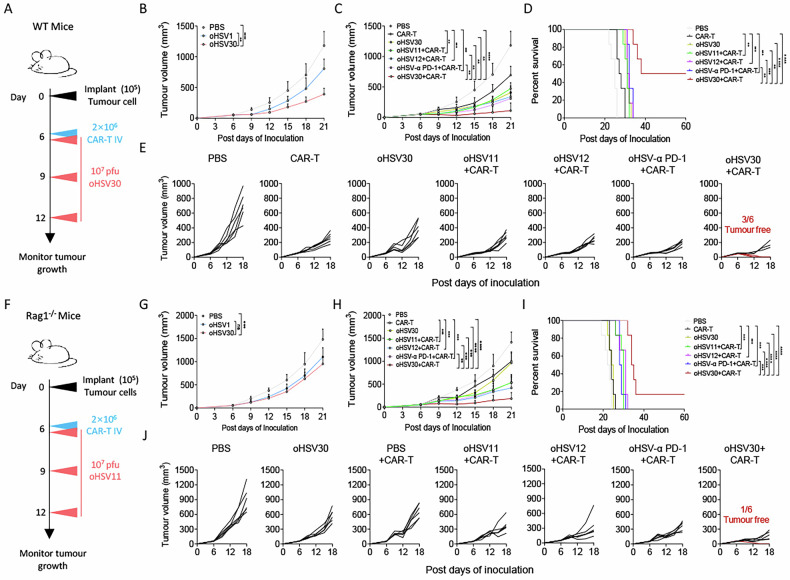
The combination of oHSV30 and CAR-T cells significantly induce tumour regression. **A**–**E** Experimental scheme. Panc2-mCD19-HVEM tumour-bearing mice received a single administration of oncolytic viruses or PBS on days 6, 9 and 12, or both three doses of oncolytic viruses (1 × 10^7^ pfu) and CAR-T cells (2 × 10^6^) on the indicated days. **B** Tumour progression was measured every 3 days during oncolytic virus monotherapy (*n* = 6 per group). Panc2-mCD19-HVEM tumour-bearing mice were treated intratumourally with oncolytic viruses or PBS on days 6, 9 and 12 and intravenous injection of PBS or CAR-T cells (2 × 10^6^) on day 6 (*n* = 6 per group). Overall tumour growth profiles (**C**), survival and tumour-free rate (**D**) and tumour growth profiles of different groups (**E**) are illustrated. **F**–**J** Experimental scheme. Panc2-mCD19-HVEM tumour-bearing mice received a single administration of oncolytic viruses or PBS on days 6, 9 and 12, or both three doses of oncolytic viruses (1 × 10^7^ pfu) and CAR-T cells (2 × 10^6^) on the indicated days (*n* = 6 per group). **G** Tumour progression was measured every 3 days during oncolytic virus monotherapy. For combination therapy, overall tumour growth profiles (**H**), survival and tumour-free rate (**I**) and tumour growth profiles of different groups (**J**) are illustrated. Data represent the mean ± SD. Statistical significance is calculated by one-way ANOVA with Tukey’s significant difference multiple comparisons (**B**, **C**, **G**, **H**). * *p* < 0.05, ** *p* < 0.01, *** *p* < 0.001, **** *p* < 0.0001.

To investigate the in vivo anti-tumour effects of combining oHSV30 with CAR-T cells, we utilised the previously described Panc2-mCD19-HVEM syngeneic tumour model (Fig. 6A). First, we observed that oHSV30 monotherapy exhibited an advantage over oHSV1 monotherapy in suppressing tumour growth in immunocompetent mice (Fig. 6B). In combination therapy, three out of six mice treated with oHSV30 plus CAR-T cells achieved complete tumour regression (CR). Although oHSV11, oHSV12 and oHSV-α PD-1 combined with CAR-T cells also partially suppressed tumour growth compared to the CAR-T cells monotherapy, no CR was observed in these groups (Fig. 6C). Correspondingly, the oHSV30 plus CAR-T cell combination significantly improved mouse survival compared to all other groups (Figs. 6D, E and S4A–C). In immunodeficient Rag1^–/–^ tumour-bearing mice, oHSV30 administration alone did not enhance the anti-tumour effect compared to oHSV1 treatment. However, oHSV30 plus CAR-T cells effectively inhibited tumour growth relative to combinations involving oHSV1, oHSV11, oHSV12, or oHSV-α PD-1 with CAR-T cells, achieving a CR rate of 1/6 (Figs. 6F–H and Fig. S4D). Survival analysis indicated that oHSV30 plus CAR-T cell treatment resulted in a significantly longer median survival compared to treatments with the other oHSV-baesd oncolytic viruses combined with CAR-T cells (Figs. 6I, J and S4E). These results collectively indicate that oHSV30 is a critical factor in enhancing the anti-tumour activity of CAR-T cells.

### oHSV30 confers enhanced infiltration, cytotoxicity and persistence to CAR-T cells against PDAC

**Fig. 7:**
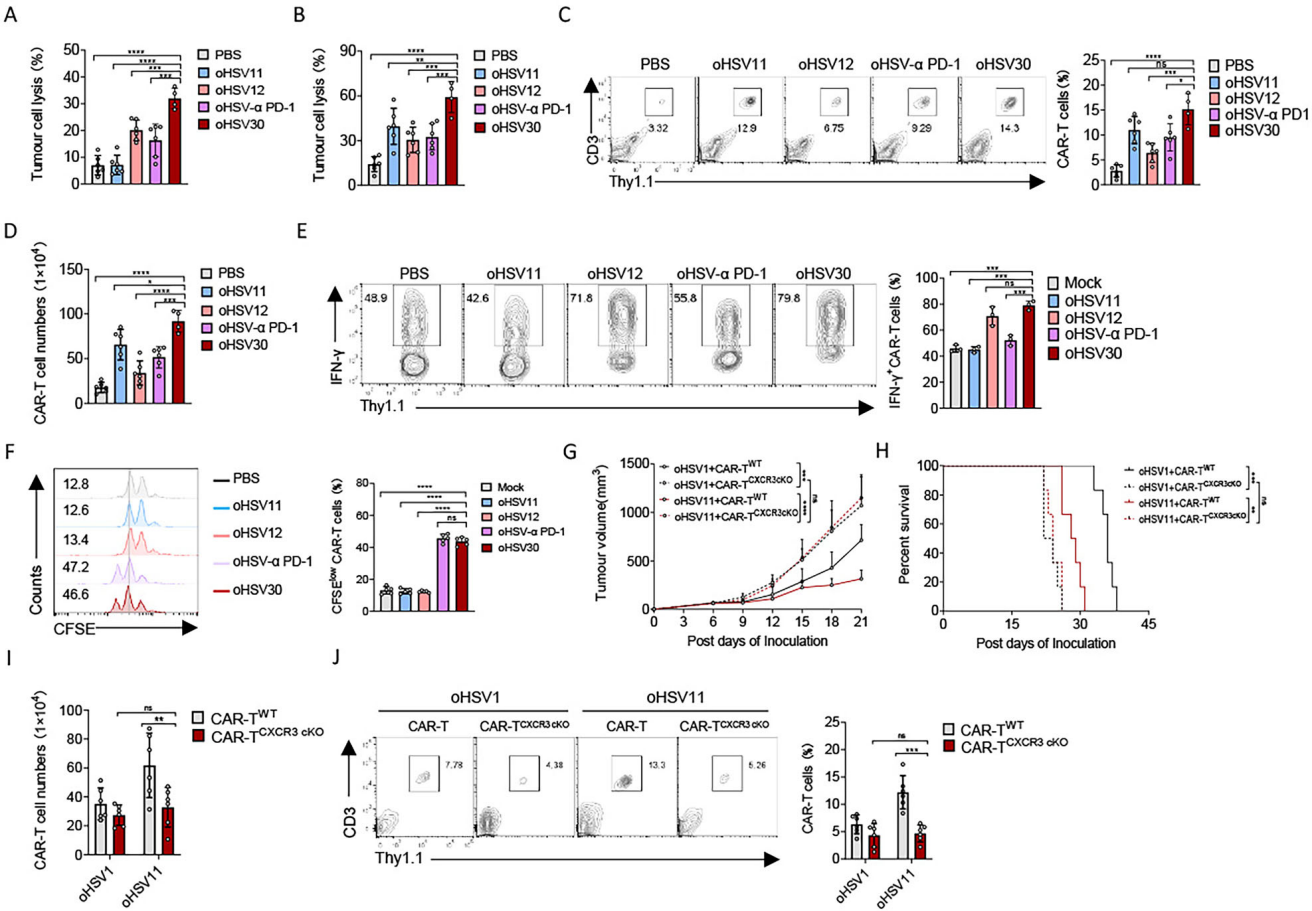
Synergistic enhancement of CAR-T cell function by oHSV30-delivered CXCL-11, IL-12 and an anti-PD-1 scFv. Panc2-mCD19-HVEM tumour-bearing Rag1^–/–^ mice were treated intratumourally with oncolytic viruses or PBS on days 6, 9 and 12 and received an intravenous injection of CAR-T cells (2 × 10^6^) on day 6. Tumour tissues were harvested from mice on day 21 after inoculation (*n* = 6 in PSB, oHSV11, oHSV12, or oHSV-α PD-1 plus CAR-T cells-treated group, *n* = 4 in oHSV30 plus CAR-T cells-treated group). Panc2-mCD19-HVEM tumour cells were co-cultured with 1 × 10^5^ tumour-infiltrated CAR-T cells (**A**) or CAR-T cells which were isolated from an equivalent weight of tumour tissue (**B**) in a 96-well flat-bottom plate. The lysis of tumour cells was measured after 36 h. The infiltration of CAR-T cells in tumour tissue (**C**, **D**), the production of IFN-γ (**E**) and proliferation (**F**) of CAR-T cells were analyzed by flow cytometry. Panc2-mCD19-HVEM tumour-bearing Rag1^–/–^ mice were treated intratumourally with oncolytic viruses or PBS on days 6, 9 and 12 and received an intravenous injection of 2 × 10^6^ CAR-T (CAR-T^WT^) cells or CXCR3-deficient CAR-T (CAR-T^CXCR3cKO^) cells on day 6 (*n* = 6 per group). Tumour progression (**G**) and survival curve (**H**) were measured on the indicated days. **I**, **J** Tumour-infiltrating CAR-T cells were analyzed by flow cytometry on day 21 after inoculation (*n* = 6 per group). Data represent the mean ± SD. Statistical significance is calculated by one-way ANOVA with Tukey’s significant difference multiple comparisons (**A**–**F**) or two-way ANOVA with Tukey’s significant difference multiple comparisons (**G**, **I**, **J**). ns not significant, *p < 0.05, **p < 0.01, ***p < 0.001, ****p < 0.0001.

To determine whether the enhanced anti-tumour effect of oHSV30 treatment depends on CAR-T cells, we performed an in vitro cytotoxicity assay to evaluate its impact on the lytic function of tumour-infiltrating CAR-T cells. The results showed a significant reduction in tumour cell numbers when co-cultured with CAR-T cells isolated from tumours treated with the oHSV30 plus CAR-T combination (Fig. 7A, B). To better understand this observed tumour-control advantage of the combinatorial treatment of oHSV30 plus CAR-T cells, flow cytometry analysis confirmed that oHSV30 treatment significantly enhanced the infiltration, cytotoxicity and persistence of CAR-T cells in situ. This was evidenced by an increased frequency and absolute number of tumour-infiltrating CAR-T cells, the highest proportion of IFN-γ-producing CAR-T cells and improved proliferative capacity (Figs. 7C–F and S4F–H). In summary, oHSV30 leverages distinct functional modules to potentiate CAR-T cell therapy: the expressed CXCL-11 mediates recruitment and infiltration of CAR-T cells into the TME, while the co-expressed IL-12 and α PD-1 scFv further augment their local cytotoxicity and persistence. Together, the combination of CXCL-11, IL-12 and α PD-1 scFv within oHSV30 dramatically enhances anti-tumour efficacy by synergistically amplifying multiple facets of the CAR-T cell response.

Prior studies have established that CXCR3⁺ classical monocytes can bolster CAR-T cell expansion and augment antitumour efficacy [22, 23]. In Panc2-mCD19-HVEM tumour-bearing mice, oHSV11 treatment increased the influx of CXCR3⁺ classical monocytes into tumours; however, this did not significantly enhance the proliferative capacity of tumour-infiltrating CAR-T cells (Fig. S5A–C). We reasoned that the limited number and proportion of infiltrating CXCR3⁺ classical monocytes in tumour tissue might underlie this result. Supporting this, in vitro co-culture assays demonstrated that higher ratios of CXCR3⁺ monocytes effectively potentiated CAR-T cell expansion and tumour cell killing (Fig. S5D, E). Thus, strategies to enhance recruitment of CXCR3⁺ monocytes may improve CAR-T cell therapeutic outcomes. To test whether oncolytic virus-derived CXCL-11 promotes CAR-T cell infiltration via CXCR3, we employed CXCR3-deficient CAR-T cells (CAR-T^CXCR3cKO^ cells) in Rag1^–/–^ mice bearing PDAC tumours. Mice were treated with either oHSV1 or oHSV11 in combination with either CAR-T^WT^ cells or CAR-T^CXCR3cKO^ cells. Comparison between oHSV1 and oHSV11 groups, both receiving CAR-T^CXCR3cKO^ cells showed no significant differences in tumour growth or overall survival (Fig. 7G, H). Moreover, CAR-T cell infiltration into the tumour microenvironment was markedly reduced in mice treated with oHSV11 plus CAR-T^CXCR3cKO^ cells relative to those receiving oHSV11 with CAR-T^WT^ cells (Fig. 7I, J). These findings confirm that CXCL-11-expressing oncolytic virus promoted robust antitumour responses primarily by facilitating CAR-T cell infiltration via the CXCL-11-CXCR3 axis, rather than by enhancing CAR-T cell cytotoxicity.

### Human CXCL-11, IL-12 and α PD-1 scFv delivered by oHSV-h30 enhance the therapeutic activity of human CAR-T cells in vivo

**Fig. 8:**
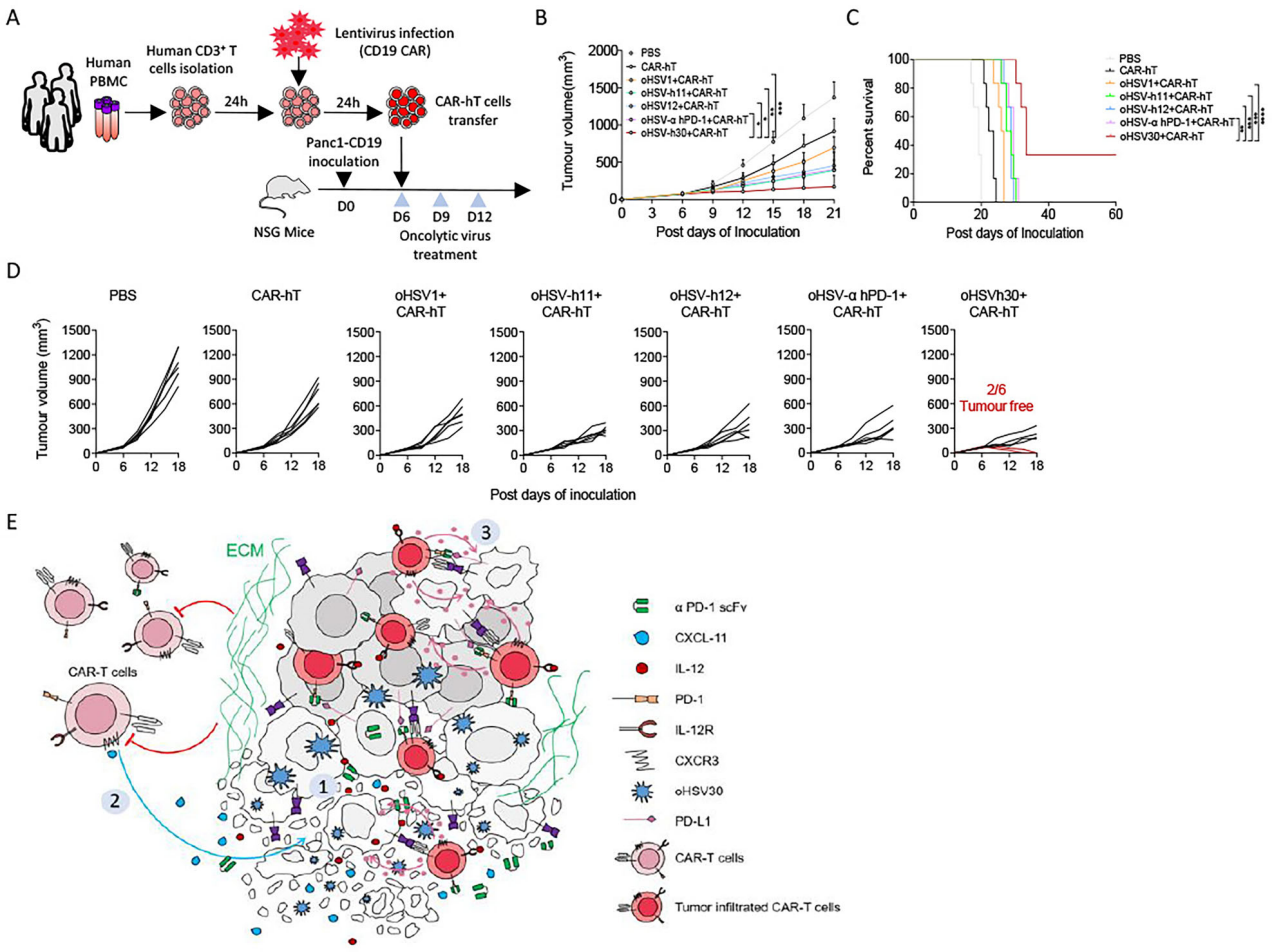
The oHSV-h30 combined with human CAR-T cells improved the therapeutic efficacy in PDAC tumour model. **A**–**D** Experimental scheme. In a subcutaneous xenograft model, Panc1-CD19 tumour-bearing NSG mice were treated with CAR-T cells, oHSV1+CAR-T, oHSV-h11+CAR-T, oHSV-h12+CAR-T, oHSV-α hPD-1 + CAR-T, or oHSV-h30+CAR-T on the indicated days. Tumour growth (**B**), survival (**C**) and tumour growth profiles of different groups (**D**) were measured over 60 days (*n* = 6 per group). **E** Schematic illustration of the engineered oncolytic virus oHSV30 to enhanced the anti-tumour responses of CAR-T cells. ① oHSV30 cleaves tumour cells and breaks through the extracellular matrix (ECM) barriers. ② CXCL-11 promotes the migration and infiltration of CAR-T cells into the tumour microenvironment TME. ③ IL-12 and α PD-1 scFv strengthened the ability of CAR-T cells to lyse tumour cells. Data represent the mean ± SD. Statistical significance is calculated by one-way ANOVA with Tukey’s significant difference multiple comparisons (**B**). * *p* < 0.05, ** *p* < 0.01, *** *p* < 0.001, **** *p* < 0.0001.

To translate our findings from murine to human systems, we first constructed oHSV-h30, an oncolytic virus expressing human CXCL-11, IL-12 and a single-chain antibody against human PD-1 (α hPD-1 scFv). We then evaluated the combined efficacy of oHSV-h30 and human CD19-targeting CAR-T cells using an immunocompromised NSG mouse model bearing CD19-overexpressing Panc1 tumours. As outlined in Fig. 8A, established tumours were treated with a single infusion of either PBS (control) or anti-CD19 CAR-T cells on day 6, followed by injections of different oncolytic viruses on days 6, 9 and 12. In groups treated with CAR-T cells alone or in combination with oHSV1, oHSV-h11, oHSV-h12, or oHSV-α hPD-1, all tumour-bearing mice succumbed to the tumour burden within 33 days. By contrast, the combination of oHSV-h30 and CAR-T cells induced complete tumour rejection in two out of six mice, which remained tumour-free throughout the two-month observation period (Fig. 8B–D). Consequently, this combination therapy significantly prolonged survival, highlighting its transformative potential. Given the current clinical challenges of applying CAR-T therapy to solid tumours, our combinatorial strategy, employing the engineered multifunctional virus oHSV-h30 as a bioenhancer for CAR-T cells, represents a promising new direction for future cancer immunotherapies (Fig. 8E).

## Discussion

CAR-T therapy has revolutionised the treatment of hematological malignancies, yet its efficacy in solid tumours remains challenging. Key obstacles include poor tumour migration and infiltration, modest cytotoxic activity and short persistence of CAR-T cells [24]. In particular, PDAC is considered an immunologically ‘cold’ tumour, which has extremely hostile conditions of the TME for CAR-T cells [25]. Oncolytic viruses act through direct tumour lysis and reprogramming of the immunosuppressive tumour microenvironment to an immunologically active state [26, 27]. Intratumoural administration of the oncolytic virus disrupted the physical barrier to immune cell infiltration, thereby enhancing the response rate and promoting tumour regression when combined with CAR-T cell therapy in solid tumours [28, 29]. Moreover, oncolytic viruses engineered to express immunomodulators are expected to enhance anti-tumour immune responses in the TME and a number of these agents have progressed to clinical evaluation across multiple cancer types [30].

Insufficient migration of CAR-T cells into the tumour reflects an unfavourable chemokine gradient, which is insufficient for their recruitment [26]. We performed a chemotaxis assay to identify the ligand with the most potent chemotactic effect on CAR-T cells and found that CXCL-11 induced significantly stronger migration. This aligns with previous studies demonstrating that CXCL-11 has a higher affinity for CXCR3 than either CXCL-9 or CXCL-10, resulting in more robust calcium flux and receptor desensitization. To enhance CAR-T cell trafficking, we generated a genetically engineered oncolytic herpes virus (oHSV11) that expresses CXCL-11 and evaluated its synergistic effect with CAR-T cells in PDAC tumour-bearing mice. We observed that intratumoural injection of oHSV11 promoted CAR-T cell infiltration more effectively than oHSV1. The additional expression of CXCL-11 by oHSV11 further enhanced the intratumoural migration of CAR-T cells, leading to a substantially greater improvement in anti-tumour efficacy relative to the combination of oHSV1 with CAR-T cells.

IL-12 is a heterodimeric pro-inflammatory cytokine that induces the production of IFN-γ, favours the differentiation of Th1 and Tc1 cells and enhances T and NK cell toxicity [31]. While arming CAR-T cells with IL-12 can boost therapeutic efficacy, it is associated with a heightened risk of triggering cytokine release syndrome (CRS) [32]. To mitigate this risk while harnessing the benefits of IL-12, we employed an oncolytic herpes virus for its localized expression. Specifically, we engineered oHSV12 to express IL-12. In combination with CAR-T cells, this virus promoted IFN-γ production and enhanced anti-tumour efficacy both in vitro and in vivo. Consistent with these findings, cytotoxicity analysis of tumour-infiltrating CAR-T cells from different treatment groups demonstrated that CAR-T cells exhibited superior anti-tumour activity when combined with oHSV12.

The anti-tumour efficacy of CAR-T cell therapy can be enhanced by blocking PD-1, a key inhibitory receptor that is highly expressed on exhausted T cells and dampens their activity [33]. To counteract PD-1-mediated suppression in CAR-T cells, a major therapeutic focus has been the development of combination therapies with PD-1 blockade, aiming to restore anti-tumour activity and promote CAR-T cell persistence [34]. By employing an oncolytic virus expressing a PD-1 scFv (oHSV-α PD-1) in combination with CAR-T cells for PDAC treatment, we demonstrated that the virus promotes a more sustained therapeutic effect by enhancing the persistence of tumour-infiltrating CAR-T cells.

The application of CAR-T cell therapy to solid tumours is constrained by several key limitations. In this study, we first confirmed that the secretion of CXCL-11, IL-12, or an α PD-1 scFv from engineered oncolytic viruses (oHSV11, oHSV12, or oHSV-α PD-1, respectively) enhanced the infiltration, cytotoxicity, or persistence of CAR-T cells. Building on these findings, we developed a multi-armed oncolytic virus, oHSV30, designed to express CXCL-11, IL-12 and an α PD-1 scFv simultaneously. This triple-modification significantly enhanced the anti-tumour efficacy of CAR-T cells against PDAC. In both immunocompetent and immunodeficient PDAC tumour-bearing mice, oHSV30 conferred more potent CAR-T cell infiltration, cytotoxicity and proliferative capacity concurrently compared to the single-factor viruses (oHSV11, oHSV12, or oHSV-α PD-1). Our results underscore the point that oHSV30 could be used as an adjuvant for CAR-T cell therapy. However, the interaction between oncolytic viruses and the tumour immune microenvironment is critical. Further investigation is required to elucidate how oHSV30, engineered to express three immunomodulators, reshapes the immune landscape when combined with CAR-T cell therapy for PDAC. Moreover, CAR-T cell therapy for solid tumours is often constrained by the complex immunosuppressive mechanisms within the tumour microenvironment. Engineering oncolytic viruses to express more immunomodulators offers a promising approach to synergise with and enhance CAR-T cell function. It is therefore imperative to further develop oncolytic viral vectors to increase their capacity for delivering multiple therapeutic genes.

In conclusion, our study demonstrates that combining multi-armed oncolytic virotherapy with CAR-T cells significantly amplifies anti-tumour efficacy. Specifically, we highlight the synergistic role of oHSV30-delivered CXCL-11, IL-12 and α PD-1 scFv in enhancing CAR-T cell function. These findings offer valuable insights for developing improved combination immunotherapies for PDAC.

## Supplementary information

Supplementary Figure

Supplementary Table S1

## Data Availability

The datasets generated and/or analyzed during the present study are available from the corresponding author on reasonable request.

## Code availability

The datasets generated and/or analyzed during the present study are available from the corresponding author on reasonable request.
