# Supplementary material for: Arming oncolytic herpes simplex virus with CXCL-11, IL-12 and a single-chain antibody against PD-1 to enhance CAR-T cell therapy in pancreatic ductal adenocarcinoma
Source: Cell Death Dis. 2026 Apr 4;17(1):455. doi: 10.1038/s41419-026-08695-0 (PMC13184102; doi:10.1038/s41419-026-08695-0)
Supplement: Supplementary file 1 — Supplementary Figure [file 41419_2026_8695_MOESM1_ESM.pdf]

Supplementary Figure 1

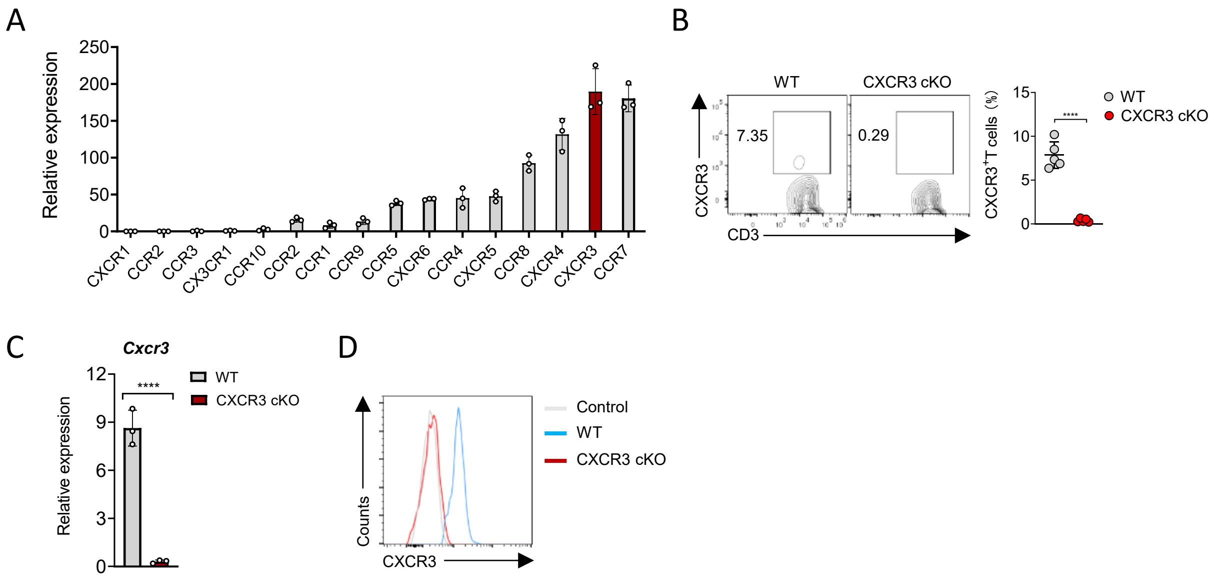

Figure S1. A. The expression of chemokine receptors in human CAR-T cells was quantified by quantitative PCR (n=3 experiments). B. Lymphocytes were isolated from the lymph nodes of $CXCR3^{fl/fl}$ (WT) or $CD4^{Cre}CXCR3^{fl/fl}$ (CXCR3 cKO) mice, and the frequency of $CXCR3^{+}CD3^{+}$T cells was monitored by flow cytometry (n = 5 per group). C-D. The expression of CXCR3 in murine CAR-T cells and CXCR3-deficient CAR-T cells was detected by qRT-PCR (C) and flow cytometry (D). Data represent the mean ± SD. Statistical significance is calculated by student's t-test. ****$p < 0.0001$.

Supplementary Figure 2

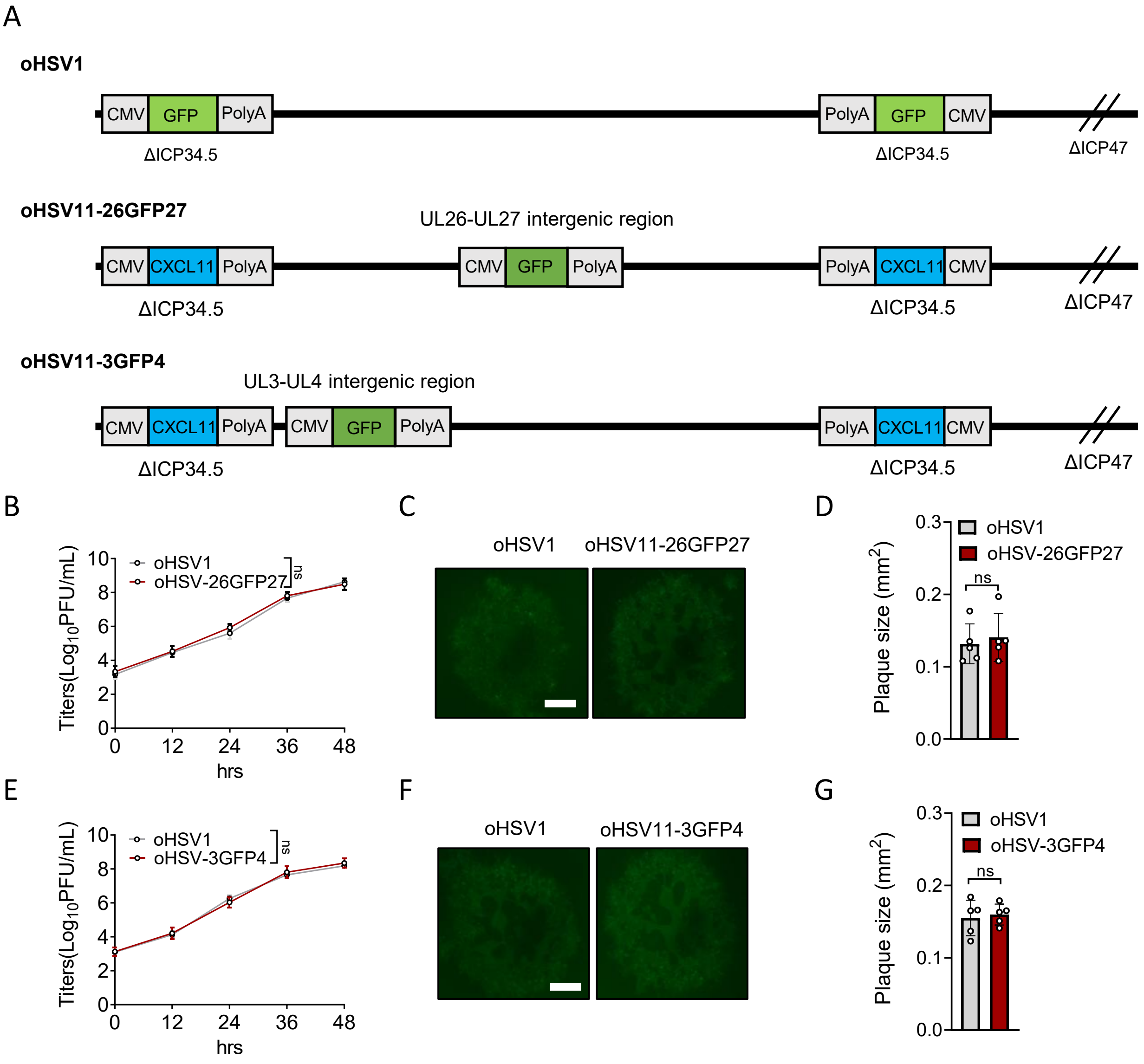

Figure S2. Use of UL26–UL27 and UL3-UL4 intergenic regions for insertion of foreign DNA into HSV.

A. Scheme showing the examination of the intergenic regions between UL-26 and UL-27 or between UL-3 and UL-4 in oHSV11. B-D. Vero cells were infected with oHSV1 and oHSV-26GFP27 at an MOI of 1, and titers were determined on Vero cells at 48 hours post-infection (n=3 experiments). GFP Plaques of oHSV1 and oHSV-26GFP27 on Vero cells under semi-solid media were similar (scale bar at 200 μm). E-G. Vero cells were infected with oHSV1 and oHSV-3GFP4 at an MOI of 1, and titers were determined on Vero cells at 48 hours post-infection (n=3 experiments). GFP Plaques of oHSV1 and oHSV-3GFP4 on Vero cells under semi-solid media were similar (scale bar at 200 μm). Data are presented as mean values ±SD. Statistical significance is calculated by student's t-test. ns= no significant.

Supplementary Figure 3

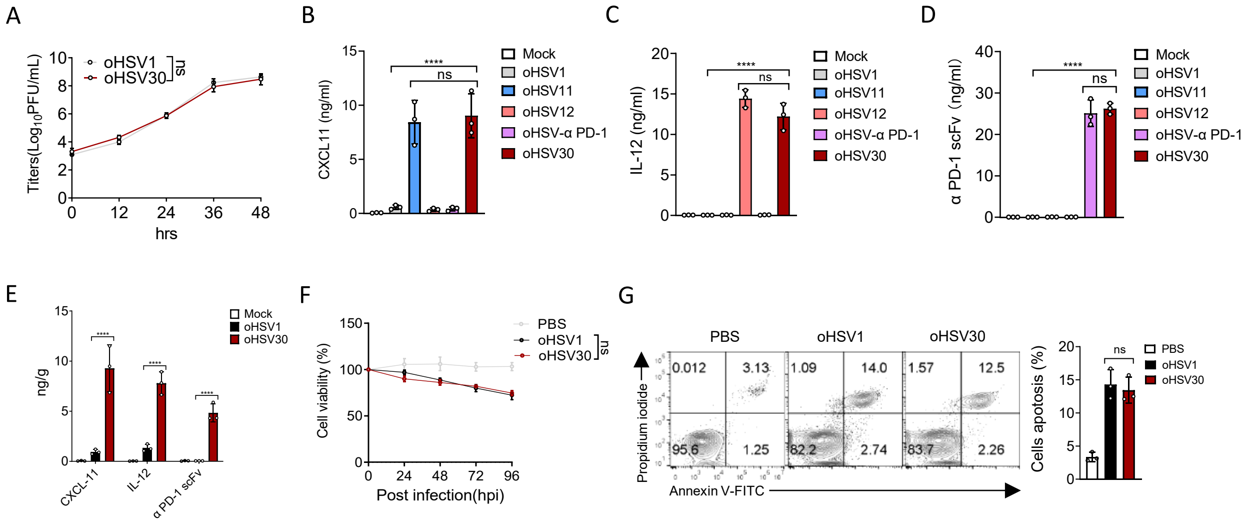

Figure S3. Construction and characterization of the oncolytic virus oHSV30.

A. 293T cells were infected with oHSV1 and oHSV30 at an MOI of 1, and titers were determined on Vero cells at 48 hours post-infection. B-D. Identification of CXCL-11, IL-12, and α PD-1 scFv expression in 293T cells infected with oncolytic viruses at an MOI of 1 for 48 hours. CXCL-11, IL-12, and α PD-1 scFv secretion in the medium was detected by ELISA (n=3 experiments). E. Panc2-mCD19-HVEM tumour-bearing mice were treated intratumourally with oncolytic viruses or PBS on day 6. Tumour tissues were isolated after 48 hours of oncolytic virus treatment, and the expression of CXCL-11, IL-12, and α PD-1 scFv was measured by ELISA (n=3 per group). F. Cell viability was measured by CCK8 assay after infection with different viruses at an MOI of 1. Uninfected cells were considered as 100% viable (n=3 experiments). G. Panc1 cell apoptosis induced by different virus was analyzed by Annexin V-FITC and PI staining (n=3 experiments). Data represent the mean ± SD. Statistical significance is calculated by one-way ANOVA with Tukey's significant difference multiple comparisons. ns, not significant, *p < 0.05, **p < 0.01, ***p < 0.001, ****p < 0.0001.

Supplementary Figure 4

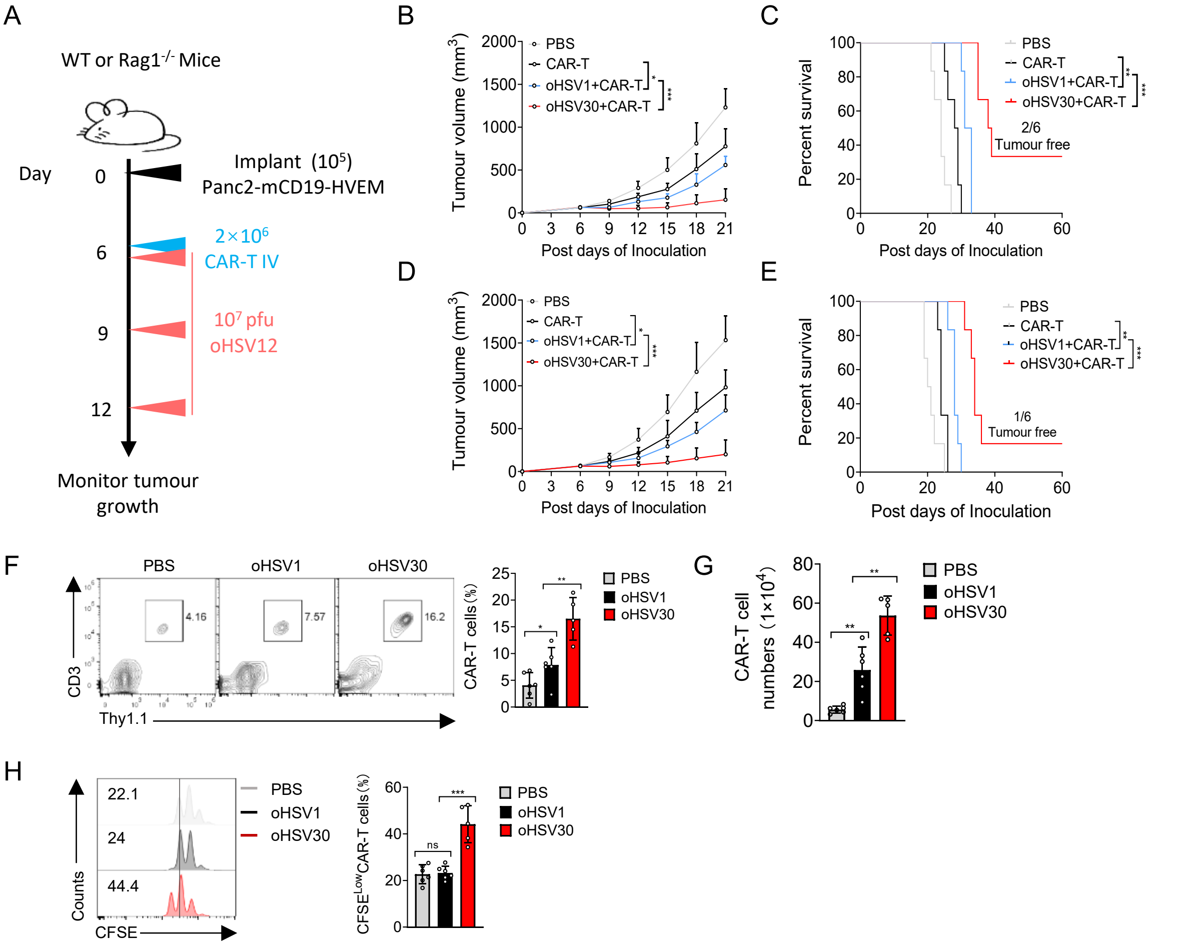

Figure S4. oHSV30 enhanced the anti-tumour efficiency.

A-E. Experimental scheme. B-C. Panc2-mCD19-HVEM tumour-bearing mice were treated intratumourally with oncolytic viruses or PBS on days 6, 9, and 12, and received an intravenous injection of PBS or CAR-T cells (2×10^6) on day 6 (n=6 per group). Tumour progression was measured every 3 days after the first treatment. A Kaplan-Meier survival curve was used to analyze the survival of tumour-bearing. D-E. Panc2-mCD19-HVEM tumour-bearing Rag1$^{-/-}$ mice were treated intratumourally with oncolytic viruses or PBS on days 6, 9, and 12, and received an intravenous injection of PBS or CAR-T cells (2×10^6) on day 6. Tumour progression (D) and survival curve (E) were measured on the indicated days. F-H. Panc2-mCD19-HVEM tumour-bearing Rag1$^{-/-}$ mice were treated intratumourally with indicated oncolytic viruses or PBS on days 6, 9, and 12, and received an intravenous injection of CAR-T cells (2×10^6) on day 6. Tumour tissues were harvested from mice on day 21 after inoculation (n=6 in PSB+CAR-T cells or oHSV1+CAR-T cells-treated group, n=5 in oHSV30+CAR-T cells-treated group). F-G. The frequency (F) and number (G) of tumour-infiltrating T cells were analyzed by flow cytometry at day 21 after inoculation. H. Panc2-mCD19-HVEM tumour cells were co-cultured with 1×10^5 tumour-infiltrating CAR-T cells with CFSE staining in a 96-well flat-bottom plate. The proliferation of CAR-T cells was measured after 36 hours. Data represent the mean ± SD. Statistical significance is calculated by one-way ANOVA with Tukey's significant difference multiple comparisons. ns, not significant, *$p < 0.05$, **$p < 0.01$, ***$p < 0.001$, ****$p < 0.0001$.

Supplementary Figure 5

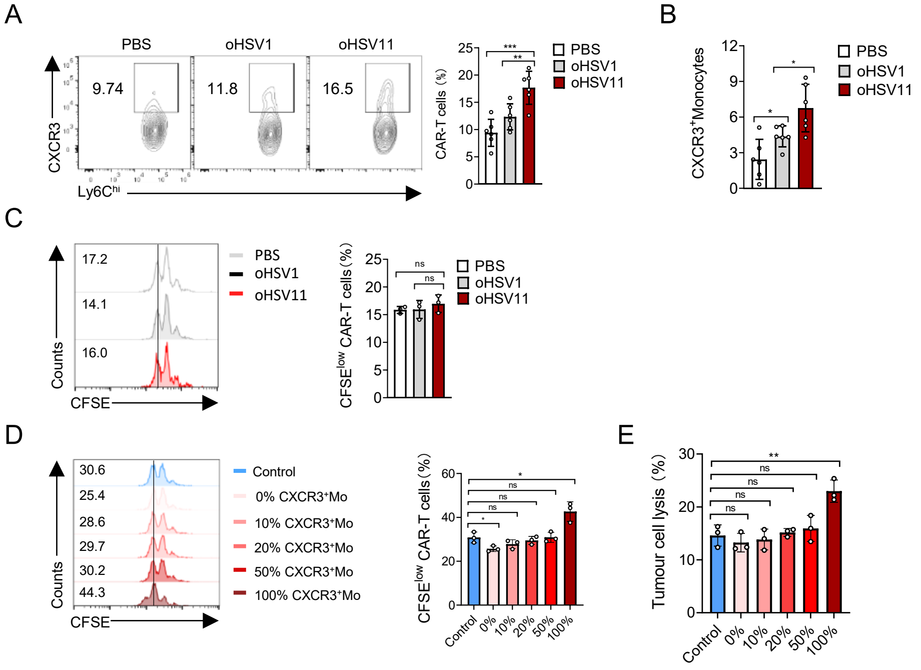

Figure S5. Increasing the frequency of CXCR3⁺ classical monocytes enhances the anti-tumour efficacy of CAR-T therapy against PDAC.

A-C. Panc2-mCD19-HVEM tumour-bearing Rag1$^{-/-}$ mice were treated intratumourally with oncolytic viruses or PBS on days 6, 9, and 12, and received an intravenous injection of PBS or CAR-T cells (2×10^6) on day 6. A-B. Tumour-infiltrating T cells were analyzed by flow cytometry on day 21 after inoculation (n=6 per group). C. Panc2-mCD19-HVEM tumour cells were co-cultured with 1×10^5 tumour-infiltrating CAR-T cells with CFSE staining in a 96-well flat-bottom plate. The proliferation of CAR-T cells were measured after 36 hours (n=3 per group). D-E. CXCR3$^+$ and CXCR3$^-$ classical monocytes (CD11b$^+$CD11c$^-$Ly6C^{hi}) were sorted from mouse PBMCs and mixed at ratios of 0%, 10%, 20%, 50%, or 100%. Panc2-mCD19-HVEM cells were co-cultured with CAR-T cells in the presence of the mixed monocytes. The proliferation of CAR-T cells (D) and killing of Panc2-mCD19-HVEM cells (E) were assessed at 48 h (n=3 per group). Data represent the mean ± SD. Statistical significance is calculated by one-way ANOVA with Tukey's significant difference multiple comparisons. ns, not significant, *p < 0.05, **p < 0.01, ***p < 0.001.
